# A molecular mechanism of mitotic centrosome assembly in *Drosophila*

**DOI:** 10.7554/eLife.03399

**Published:** 2014-08-22

**Authors:** Paul T Conduit, Jennifer H Richens, Alan Wainman, James Holder, Catarina C Vicente, Metta B Pratt, Carly I Dix, Zsofia A Novak, Ian M Dobbie, Lothar Schermelleh, Jordan W Raff

**Affiliations:** 1 Sir William Dunn School of Pathology, University of Oxford, Oxford, United Kingdom; 2 Medical Research Council Laboratory of Molecular Biology, Cambridge, United Kingdom; 3 Oxford Micron advanced imaging unit, Department of Biochemistry, University of Oxford, Oxford, United Kingdom; The Gurdon Institute, United Kingdom

**Keywords:** centrosome, PCM, centrosome maturation, live super-resolution, FRAP, *D. melanogaster*

## Abstract

Centrosomes comprise a pair of centrioles surrounded by pericentriolar material (PCM). The PCM expands dramatically as cells enter mitosis, but it is unclear how this occurs. In this study, we show that the centriole protein Asl initiates the recruitment of DSpd-2 and Cnn to mother centrioles; both proteins then assemble into co-dependent scaffold-like structures that spread outwards from the mother centriole and recruit most, if not all, other PCM components. In the absence of either DSpd-2 or Cnn, mitotic PCM assembly is diminished; in the absence of both proteins, it appears to be abolished. We show that DSpd-2 helps incorporate Cnn into the PCM and that Cnn then helps maintain DSpd-2 within the PCM, creating a positive feedback loop that promotes robust PCM expansion around the mother centriole during mitosis. These observations suggest a surprisingly simple mechanism of mitotic PCM assembly in flies.

**DOI:** http://dx.doi.org/10.7554/eLife.03399.001

## eLife digest

Long protein filaments called microtubules perform a range of roles inside cells—for example, they give the cell its shape and help to divide its genetic material during cell division. In animal cells, microtubules emerge from structures called centrosomes. These contain two cylindrical structures called centrioles that are surrounded by a matrix of pericentriolar material made from several hundred different proteins. Problems with centrosomes have been linked to several disorders, including cancer.

In the fruit fly *Drosophila*, it was long thought that the pericentriolar material assembles on an underlying ‘scaffold’, the composition of which had remained unclear. A protein called Centrosomin was a good candidate molecule, as it is required to maintain the proper structure of the pericentriolar material. In addition, Centrosomin molecules continuously spread away from the centrioles into the matrix providing a clear centriole–matrix connection. However, if Centrosomin is not present in a cell, some protein is still recruited around the centrioles. Conduit et al. therefore suspected that Centrosomin works together with another protein to build the scaffold.

Conduit et al. used super-resolution microscopy to observe the behaviour of several proteins, thought most likely to help Centrosomin to form the scaffold. Only one, called DSpd-2, builds outwards from the centrioles like Centrosomin. Genetic tests showed that both Centrosomin and DSpd-2 are important for the other proteins to localize to the pericentriolar material. If one of either Centrosomin or DSpd-2 is missing from the cell, reduced amounts of protein are recruited around the centrioles but the matrix still partially forms. Without both proteins, however, the matrix does not form at all.

Conduit et al. found that a third protein helps to recruit Centrosomin and DSpd-2 to the older of the two centrioles (also known as the mother centriole). DSpd-2 then draws in more Centrosomin. As Centrosomin helps to hold the DSpd-2 proteins in the pericentriolar material, this enables even more Centrosomin to be recruited, and so forms a positive feedback loop that helps the scaffold to continue growing.

The findings of Conduit et al. provide a simple mechanism for building the scaffold that supports the formation of the centrosome in the fruit fly *Drosophila*. Whether a similar mechanism is used to construct centrosomes in other species remains to be investigated.

**DOI:** http://dx.doi.org/10.7554/eLife.03399.002

## Introduction

Centrosomes help regulate many cell processes, including cell shape, cell polarity, and cell division (Doxsey et al., 2005; Bettencourt-Dias and Glover, 2007), and centrosome defects have been implicated in several human pathologies (Nigg and Raff, 2009; Zyss and Gergely, 2009). Centrosomes are the major microtubule (MT)-organising centres (MTOCs) in many animal cells. They form when centrioles assemble a matrix of pericentriolar material (PCM) around themselves. Several hundred proteins are concentrated in the PCM, including many MT-organising proteins, cell-cycle regulators, and checkpoint and signalling proteins (Müller et al., 2010); thus, the centrosome appears to function as an important co-ordination centre in the cell (Doxsey et al., 2005). Although centrioles usually organize only small amounts of PCM in interphase cells, the PCM expands dramatically as cells prepare to enter mitosis—a process termed centrosome maturation (Khodjakov and Rieder, 1999).

Many proteins have been implicated in mitotic PCM assembly. These include (1) centriole-associated proteins, such as ‘Asl/Cep152’ (Bonaccorsi et al., 1998; Varmark et al., 2007; Dzhindzhev et al., 2010) and ‘Sas-4/CPAP’ (Cho et al., 2006; Gopalakrishnan et al., 2011), (2) proteins that have a centriole-associated fraction and a fraction that spreads out into the PCM, such as ‘Pericentrin/D-PLP’ (Martinez-Campos et al., 2004; Zimmerman et al., 2004; Fu and Glover, 2012; Lawo et al., 2012; Mennella et al., 2012) and ‘DSpd-2/Cep192’ (Pelletier et al., 2004; Dix and Raff, 2007; Gomez-Ferreria et al., 2007; Giansanti et al., 2008; Zhu et al., 2008; Joukov et al., 2010; Decker et al., 2011; Joukov et al., 2014), (3) proteins that reside in the PCM, such as ‘Cnn/Cdk5Rap2’ (Megraw et al., 1999; Lucas and Raff, 2007; Fong et al., 2008; Barr et al., 2010; Conduit et al., 2010), ‘DGp71WD/NEDD1’ (Haren et al., 2006, 2009; Lüders et al., 2006; Manning et al., 2010), and ‘γ-tubulin’ (Sunkel et al., 1995; Hannak et al., 2002), and (4) mitotic protein kinases, such as ‘Polo/Plk1’ and ‘Aurora A’ (Barr and Gergely, 2007; Petronczki et al., 2008). In recent super-resolution microscopy studies, several of these proteins appeared to be highly organized around interphase centrioles, but the organisation of proteins within the extended mitotic PCM was much less apparent (Fu and Glover, 2012; Lawo et al., 2012; Mennella et al., 2012; Sonnen et al., 2012).

It has long been thought that the mitotic PCM is assembled on an underlying scaffold structure (Dictenberg et al., 1998; Schnackenberg et al., 1998). We recently showed that *Drosophila* Centrosomin (Cnn) can form such a scaffold around centrioles and that this scaffold is assembled from the inside out (Conduit et al., 2014): Cnn molecules continuously incorporate into the scaffold around the centrioles and the scaffold then fluxes slowly outward, away from the centrioles. This inside out assembly mechanism could be important, as it potentially allows the assembly of the mitotic PCM to be regulated by the centrioles.

Many PCM proteins, however, can be recruited to mitotic centrosomes in the absence of Cnn, albeit at reduced levels (Lucas and Raff, 2007), suggesting that at least one other protein must be able to form a scaffold around centrioles that can recruit other PCM components. We reasoned that such a scaffold might also be assembled from the inside out. To identify such a protein(s), we analyzed the dynamic behaviour of the eight *Drosophila* centrosomal proteins that, in addition to Cnn, have been most strongly implicated in mitotic PCM recruitment: Asl, Sas-4, D-PLP, DSpd-2, γ-tubulin, DGp71WD, Polo, and Aurora A. We found that only DSpd-2 behaves like Cnn, as it incorporates into the PCM close to the centrioles and then spreads slowly outward to form a scaffold-like structure that recruits other PCM components. Importantly, in the absence of either DSpd-2 or Cnn, PCM recruitment is diminished, but in the absence of both proteins, it is abolished. We show that Asl appears to initiate the recruitment of DSpd-2 and Cnn exclusively to the mother centrioles; DSpd-2 then helps to recruit more Cnn, while Cnn helps to maintain DSpd-2 within the PCM, thus creating a positive feedback loop that promotes the scaffold assembly. Thus, mitotic PCM assembly appears to be a surprisingly simple process in flies: Asl initiates the recruitment of Spd-2 and Cnn to mother centrioles, and these proteins then assemble into scaffolds that spread out from the mother centriole and form a platform upon which most, if not all, other PCM proteins ultimately assemble.

## Results

### Most PCM proteins are recruited to centrosomes by binding sites that are distributed throughout the PCM

We used spinning disk confocal microscopy to perform fluorescence recovery after photobleaching (FRAP) experiments in combination with radial-profiling to quantify the spatio-temporal dynamics of various GFP-fusion proteins in *Drosophila* syncytial embryos. We selected the nine proteins, including Cnn, which have been most strongly implicated in the PCM recruitment in flies: Asl-GFP, AurA-GFP, GFP-Cnn, DGp71WD-GFP, D-PLP-GFP, DSas-4-GFP, DSpd-2-GFP, γ-tubulin-GFP, and Polo-GFP (Mennella et al., 2013). We assessed the expression level of these fusion proteins relative to their endogenous proteins by Western blotting (Figure 1—figure supplement 1).

**Figure 1.**
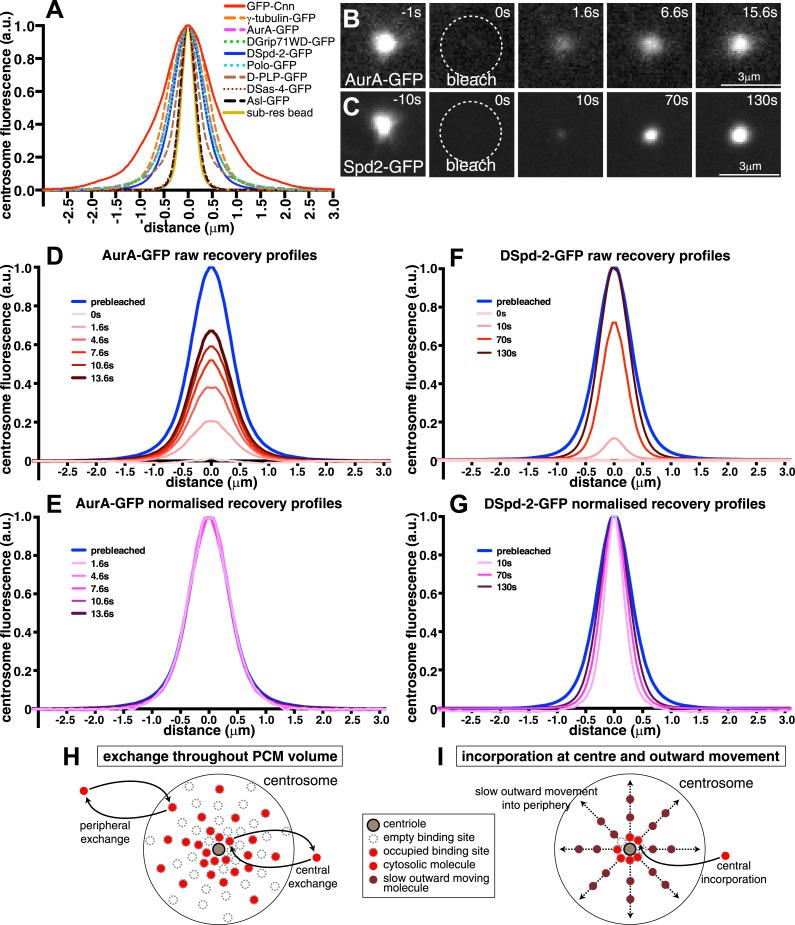
Centrosomal DSpd-2 displays an unusual dynamic behaviour. (**A**) Graph shows the centrosomal fluorescence intensity profiles of various centrosomal proteins, along with the profile of 170 nm sub-resolution beads. (**B** and **C**) Images show the FRAP behaviour of AurA-GFP (**B**) and DSpd-2-GFP (**C**); time before and after photobleaching (t = 0) is indicated. Note how AurA-GFP fluorescence appears to recover evenly throughout the region it originally occupied, whereas DSpd-2-GFP fluorescence appears to initially recover only in the centre of the PCM and then spread outward. (**D**–**G**) Quantification of the recovery dynamics of AurA-GFP (**D** and **E**) and DSpd-2-GFP (**F** and **G**). Graphs show the average fluorescence intensity profile of at least 10 centrosomes at the selected time-points after photobleaching: (**D**) and (**F**) show the pre-bleached profiles (blue lines) and successive ‘raw’ recovery profiles (various shades of red), whereas (**E**) and (**G**) show the pre-bleached profiles and successive normalized recovery profiles (various shades of pink/purple—normalized so that their peak intensity is equal to the peak intensity of the pre-bleached profile). The normalized recovery curves of AurA-GFP are essentially identical to the pre-bleached profile at all time-points (**E**), while for DSpd-2-GFP they are initially narrower and spread outward over time (**G**). (**H** and **I**) Schematics illustrate the dynamic behaviour of AurA-GFP (and most other PCM components) (**H**) and DSpd-2-GFP (and GFP-Cnn) (**I**). Cytoplasmic AurA-GFP molecules exchange with binding sites spread throughout the PCM (**H**), whereas DSpd-2-GFP molecules are recruited by binding sites located close to the centrioles; once released from these binding sites the molecules spread slowly outward into the more peripheral regions of the PCM (**I**). See also Figure 1—figure supplements 1 and 2, and Video 1. **DOI:** http://dx.doi.org/10.7554/eLife.03399.003

Prior to photobleaching, the fusion proteins displayed different centrosomal distributions (Figure 1A). Asl-GFP and DSas-4-GFP are known to be closely associated with centrioles (Fu and Glover, 2012; Mennella et al., 2012), and their fusions were tightly localized in the centre of the PCM; they exhibited a fluorescence intensity profile similar to that of sub-resolution (170 nm) beads (Figure 1A), indicating that their true distribution was below the resolution of our microscope system. The other proteins were all distributed more broadly throughout the PCM to varying extents.

**Figure 1—figure supplement 1.**
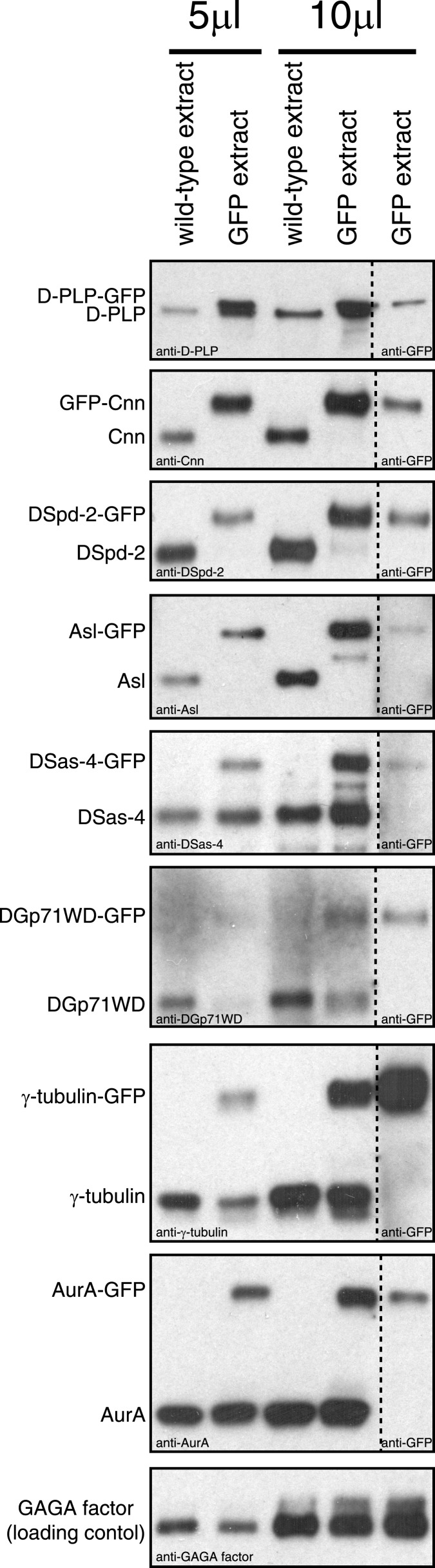
An analysis of the expression levels of several GFP-tagged centrosomal proteins. Western blots compare the levels of various centrosomal proteins (as indicated) from wild-type embryo extracts (lanes 1 and 3) or from extracts of embryos expressing a GFP-fused version of the protein (lanes 2, 4, and 5). Lanes 1 to 4 were probed with antibodies raised against the centrosomal protein in question; lane 5 was probed with anti-GFP antibodies. Lanes 1 and 2 were loaded with 5 μl of extract; lanes 3 to 5 were loaded with 10 μl of extract. The bottom panel shows a Western blot for GAGA factor, which acts as a loading control (all blots were controlled in this way, but only one example is shown here). GFP-Cnn, DSpd-2-GFP, and Asl-GFP were expressed in a null mutant background; the other GFP-fusions were expressed in a wild-type background. Note how GFP-Cnn and D-PLP-GFP appear to be expressed at slightly higher levels than the endogenous protein, but that the other fusion proteins are expressed either at similar or at slightly lower levels than the endogenous protein. We previously showed that increasing the cytoplasmic concentration of GFP-Cnn increases the rate at which it is incorporated into the PCM, but does not change the inside out incorporation behaviour (Conduit et al., 2010). Note also that GFP-Cnn and DSpd-2-GFP are overexpressed and underexpressed, respectively, but both display a similar dynamic behaviour (Figure 1F,G, Figure 1—figure supplement 2M,N), again suggesting that the expression level of a GFP-fusion protein does not significantly affect the mode in which it is incorporated into the PCM. We have not been able to analyze the relative expression level of Polo-GFP, as we were unable to get anti-Polo antibodies to work on Western blots. **DOI:** http://dx.doi.org/10.7554/eLife.03399.004

**Figure 1—figure supplement 2.**
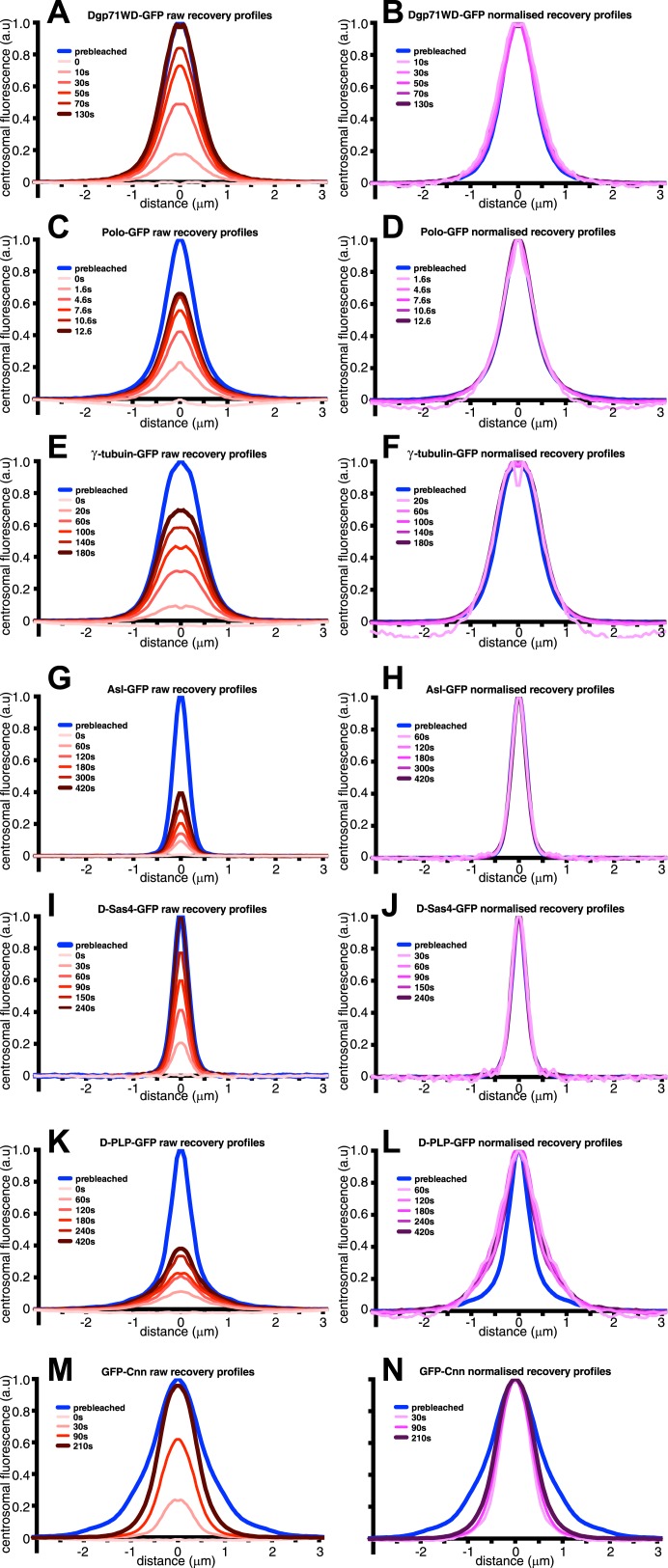
An analysis of the dynamic behaviour of several GFP-tagged centrosomal proteins. Quantification of the FRAP recovery dynamics of DGp71WD-GFP (**A** and **B**), Polo-GFP (**C** and **D**), γ-tubulin-GFP (**E** and **F**), Asl-GFP (**G** and **H**), DSas-4-GFP (**I** and **J**), D-PLP-GFP (**K** and **L**), and GFP-Cnn (**M** and **N**). Graphs show the average fluorescence intensity profile of ≥10 centrosomes (‘Materials and methods’) at selected time-points after photobleaching (t = 0). Graphs on the left of each panel display pre-bleached profiles (blue lines) and ‘raw’ recovery profiles (various shades of red lines). Graphs on the right of each panel display pre-bleached profiles and recovery profiles (various shades of purple lines) that have all been normalized so that their peak intensity is equal to the peak intensity of the pre-bleach profile. Note how the normalized recovery profiles of DGp71WD-GFP (**B**), Polo-GFP (**D**), and γ-tubulin-GFP (**F**) are very similar to their pre-bleached profiles, indicating that they are all incorporated evenly throughout the PCM domain they originally occupied. This is also true for Asl-GFP (**H**) and DSas-4-GFP (**J**), but these proteins have the same distribution as sub-resolution beads (Figure 1A), so their true distribution cannot be resolved on this microscope system. The recovery dynamics of D-PLP-GFP (**K** and **L**) are complicated by the presence of a slow-exchanging centriole fraction and a fast-exchanging PCM fraction (Martinez-Campos et al., 2004), which cannot be properly distinguished at this resolution. It appears that the slow recovery rate of the centriole fraction compared to the PCM fraction causes the normalized recovery profiles to be wider than the pre-bleached profile. It can be seen, however, that the PCM fraction of D-PLP shows no sign of outward movement through the PCM (**L**). The normalized recovery profiles of GFP-Cnn (**N**) are narrower than the pre-bleached profile and spread outwards over time, reflecting the fact that Cnn molecules are initially incorporated only in the centre of the PCM and then move slowly outward (Conduit et al., 2014). **DOI:** http://dx.doi.org/10.7554/eLife.03399.005

After photobleaching, the Asl-GFP, Sas-4-GFP, AurA-GFP, DGp71WD-GFP, γ-tubulin-GFP, and Polo-GFP fluorescence all recovered at different rates, but each protein appeared to recover evenly throughout the domain that it originally occupied (Figure 1B,D; Figure 1—figure supplement 2; Video 1A,B). This even recovery was confirmed when the recovery profiles were normalized so that their peak fluorescence intensity at each time-point equalled one; this showed that, at all time-points, the shape of each normalized recovery curve closely matched the shape of its respective pre-bleached profile (Figure 1E, Figure 1—figure supplement 2; Video 1C). We conclude that most PCM proteins are recruited to centrosomes by binding sites that are already distributed throughout the PCM volume that each protein occupies (Figure 1H). These observations strongly support the idea that most PCM proteins are recruited by an underlying scaffold structure.

**Video 1.** DSpd-2-GFP molecules are initially incorporated into the centre of the PCM. (related to Figure 1). All Videos shown here are maximum intensity projections of image stacks. These videos illustrate the dynamic behaviour of AurA-GFP (**A**–**C**) or DSpd-2-GFP (**D**–**F**) at centrosomes in *Drosophila* embryos. Time before and after photobleaching (t = 0 s) is shown at the top right of (**C**) and (**F**). The graphs in (**B**) and (**E**) are the line profiles representing the distribution of the AurA-GFP and the DSpd-2-GFP centrosomal fluorescence, respectively, through time: the blue lines represent the pre-bleached profiles, and the red lines represent the recovering profiles. Note how the AurA-GFP fluorescence signal appears to recover evenly throughout the PCM domain it originally occupied, whereas the DSpd-2-GFP fluorescence signal appears to initially recover in the centre of the PCM and then spreads outwards. This is most clearly seen in (**C**) and (**F**) where the recovery profiles at all time-points have been normalized so that their peak intensities are equal to 1 (purple lines). Note how the normalized AurA-GFP recovery profiles all closely overlap with the pre-bleached profile, whereas the normalized DSpd-2-GFP recovery profiles are all narrower than the pre-bleached profile and spread out over time. **DOI:** http://dx.doi.org/10.7554/eLife.03399.006

We note that the dynamics of D-PLP-GFP are complicated by the presence of two distinct centrosomal fractions of D-PLP: one fraction tightly localized at the centriole that turns over slowly, and another that localizes to the PCM and turns over rapidly (Martinez-Campos et al., 2004) (Figure 1—figure supplement 2K,L). Nevertheless, it was clear from recovery profiles that the PCM fraction of D-PLP-GFP did not initially recover centrally and then move outwards, but rather recovered throughout the PCM volume.

### DSpd-2-GFP behaves like GFP-Cnn and is incorporated into the PCM close to the centrioles and then spreads outward

Unlike the other PCM components we tested, DSpd-2-GFP fluorescence initially recovered only in the central region of the PCM and then gradually began to recover in the more peripheral regions (Figure 1C,F,G; Video 1D–F). This suggested that, like Cnn (Conduit et al., 2014; Figure 1—figure supplement 2M,N), DSpd-2 is recruited to binding sites that are only located close to the centrioles; once released from these sites, it then spreads slowly outward into the more peripheral regions of the PCM (Figure 1I).

**Figure 2.**
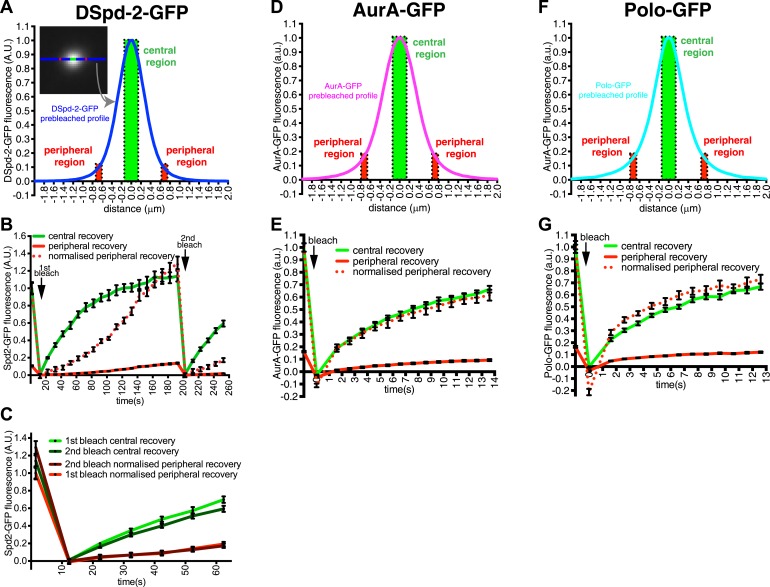
DSpd-2-GFP molecules spread away from the centrioles. (**A**) Graph displays the pre-bleached profile of DSpd-2-GFP at centrosomes in *Drosophila* embryos (blue line, average of 10 centrosomes). Boxes highlight the central region of the PCM (green box) and peripheral regions of the PCM (red boxes) that were analyzed in a FRAP experiment. (**B**) Graph displays the average fluorescence intensity through time in the centre (green line) and periphery (red and dotted red lines) of the PCM after photobleaching. Arrows indicate times of photobleaching. The dotted red line represents the peripheral recovery after it has been normalized so that its initial pre-bleached value is equal to the initial pre-bleached value of the central recovery curve. Note how the central DSpd-2-GFP fluorescence recovery exhibits a typical logarithmic-shaped FRAP curve, with a fast initial rate that slows over time. In contrast, peripheral DSpd-2-GFP fluorescence recovery exhibits a very unusual behaviour as it is initially slow and then speeds up over time. This strongly suggests that DSpd-2-GFP molecules move from the centre to the periphery of the PCM (see main text). (**C**) Graph compares the initial recovery kinetics in the central (green lines) or peripheral (red lines) regions of the PCM after a first (light lines) and then second (dark lines) photobleaching event. The second photobleaching event took place when the rate of recovery in the centre was slow and the rate of recovery in the periphery was fast (see t = 200 s in **B**). Note that after the second bleaching event the central recovery returned to its original fast rate and the peripheral recovery returned to its original slow rate, showing that the increasing rate of recovery in the periphery was not due to peripheral binding sites gradually exchanging faster over time. (**D**–**G**) Graphs display the pre-bleached profiles (**D** and **F**) and recovery kinetics (**E** and **G**) of AurA-GFP (**D** and **E**) and Polo-GFP (**F** and **G**) at centrosomes in embryos (average of 11 centrosomes) in the same format as shown for DSpd-2-GFP (**A** and **B**). Note how the peripheral recovery curves of AurA-GFP (red and dotted red lines in **E**) and Polo-GFP (red and dotted red lines in **G**) both have a similar shape to their central recovery curves, indicating that the unusual peripheral kinetics of DSpd-2-GFP (red and dotted red lines in **B**) are not observed with other proteins that have a similar distribution around the centrioles. Error bars = SEM. **DOI:** http://dx.doi.org/10.7554/eLife.03399.007

A comparison of DSpd-2-GFP recovery kinetics in the central and peripheral regions strongly supported this interpretation (Figure 2A–C). The central region of DSpd-2-GFP fluorescence exhibited a typical, logarithmic-shaped, FRAP recovery curve, with a fast initial recovery rate that gradually slowed over time (*green line*, Figure 2B). In contrast, the peripheral regions exhibited an unusual FRAP recovery curve, with a slow initial recovery rate that gradually increased over time (*solid red line*, Figure 2B); this was best seen when the peripheral recovery curve was normalized so that the pre-bleached signal equalled one (*dotted red line*, Figure 2B). These unusual recovery dynamics at the periphery of the PCM can most easily be explained if DSpd-2-GFP molecules are constantly moving from the centre of the centrosome to the periphery: as the number of fluorescent molecules in the centre increases with time after photobleaching, so the number of fluorescent molecules moving from the centre into the periphery gradually increases, explaining why the peripheral recovery rate speeds up over time.

### DSpd-2 and Cnn have partially overlapping distributions around centrioles

**Figure 3.**
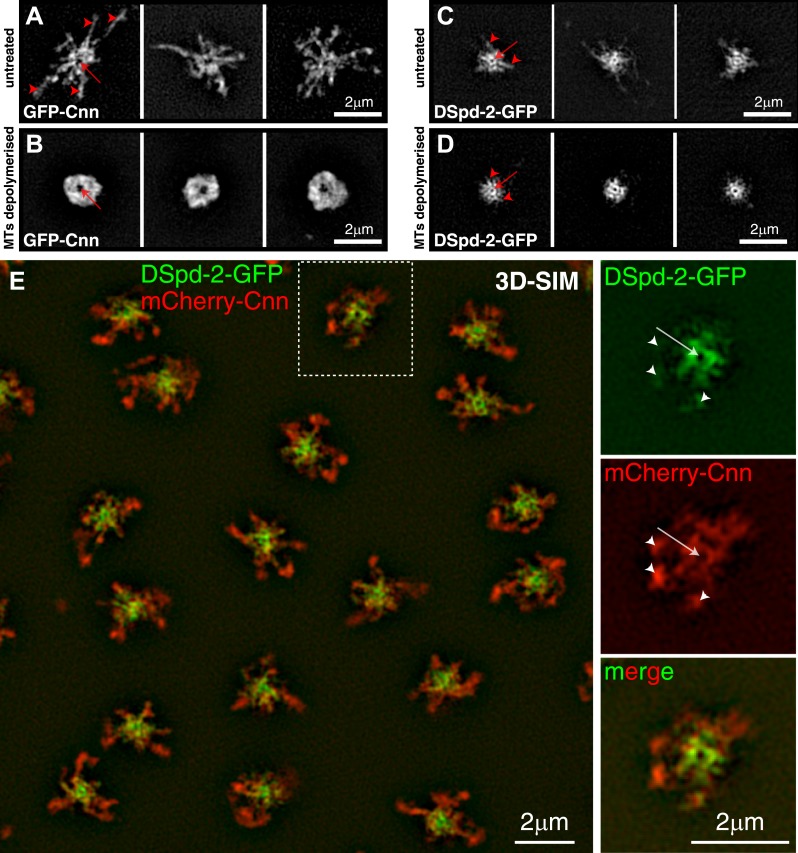
DSpd-2-GFP appears to form scaffold-like structure around the centrioles that partially co-localizes with the Cnn scaffold. (**A**–**D**) 3D-SIM images of centrosomes from embryos expressing either GFP-Cnn (**A** and **B**) or DSpd-2-GFP (**C** and **D**) where the MTs are either present (**A** and **C**) or have been depolymerized by colchicine injection (**B** and **D**). (**A**) In untreated embryos, large projections of GFP-Cnn extend outwards (red arrowheads) from a central hollow (red arrow), which presumably contains the mother centriole. (**B**) After MT depolymerisation, the GFP-Cnn scaffold collapses into a largely amorphous structure; presumably, the molecular detail of the scaffold cannot be resolved even at this high resolution. The slightly larger central ‘hollow’ in the GFP-Cnn signal (red arrow) likely reflects the ability of the Cnn molecules to move a short distance away from the centre of the centrosome in the absence of microtubules (Conduit et al., 2014); these molecules then get ‘trapped’ in the more peripheral regions of the PCM, as they cannot efficiently leave the centrosome in the absence of MTs (Conduit et al., 2014). (**C**) In untreated embryos, DSpd-2-GFP appears as a series of spoke-like projections (red arrowheads) that extend away from a central ring (red arrow), which presumably surrounds the mother centriole; some of these projections weakly extend into the peripheral PCM. (**D**) After MT de-polymerisation, DSpd-2-GFP retains a large degree of its structure: there is a clear central ring (red arrow) with several spoke-like projections extending outwards (red arrowheads); these projections, however, no longer appear to extend into the more peripheral regions of the PCM. (**E**) Two-colour 3D-SIM images of centrosomes in untreated embryos co-expressing DSpd-2-GFP (green) and mCherry-Cnn (red). The panels on the right are enlargements of the boxed centrosome in the panel on the left; note the clear hollow in the centre of the centrosome (arrows), and how the mCherry-Cnn signal extends further away from the centrioles than the DSpd-2-GFP signal, although weak DSpd-2-GFP fluorescence can often be observed in the same region as the more peripheral mCherry-Cnn (arrowheads). See also Figure 3—figure supplement 1. **DOI:** http://dx.doi.org/10.7554/eLife.03399.008

We previously used super-resolution structured illumination microscopy (3D-SIM) in living fly embryos to show that the GFP-Cnn scaffold radiates away from the centrioles and forms large projections that extend outwards along the centrosomal MTs (Conduit et al., 2014; Figure 3A). DSpd-2-GFP had a similar distribution with several spoke-like projections extending from a central ring (Figure 3C), although, in agreement with our radial profiling data, much less DSpd-2 extended into the more peripheral regions of the PCM (Figure 3C). Two-colour 3D-SIM in living embryos confirmed that DSpd-2-GFP and mCherry-Cnn extensively overlapped in the more central regions of the PCM (Figure 3E, Figure 3—figure supplement 1) and revealed that the more peripheral Cnn projections usually contained small amounts of DSpd-2 (arrowheads, Figure 3E and Figure 3—figure supplement 1B–D).

**Figure 3—figure supplement 1.**
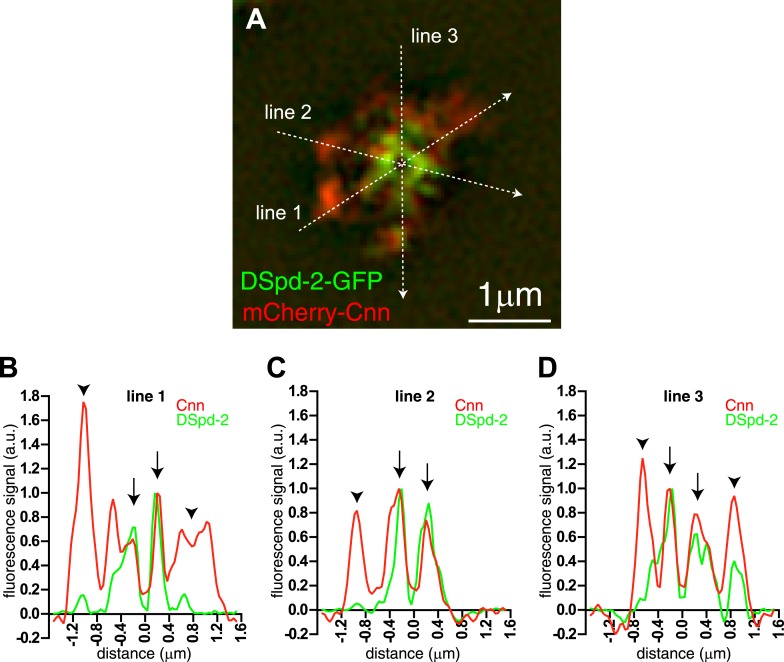
An approximate quantification of mCherry-Cnn and DSpd-2-GFP co-localisation. The mCherry-Cnn (red) and DSpd-2-GFP (green) fluorescence intensity along the three lines that run through a 3D-SIM image of a centrosome in (**A**) are plotted on the graphs in (**B**), (**C**), and (**D**). Green lines represent the fluorescent intensity of DSpd-2-GFP, and red lines represent the fluorescent intensity of mCherry-Cnn. The graphs have been normalized so that the peak value in the central region of the PCM is equal to 1. Arrows within the graphs indicate the central regions of the centrosome where mCherry-Cnn and DSpd-2-GFP extensively co-localize. Arrowheads indicate the more peripheral regions of the centrosome that contain high levels of mCherry-Cnn fluorescence but only low levels of DSpd-2-GFP fluorescence. **DOI:** http://dx.doi.org/10.7554/eLife.03399.009

Unsurprisingly, the GFP–Cnn projections (Figure 3A) were no longer visible when the MTs were depolymerized with colchicine, and the GFP–Cnn scaffold became more compact and amorphous in appearance (Figure 3B). The small amounts of DSpd-2 extending into the peripheral PCM (Figure 3C) were also lost when the MTs were depolymerized (Figure 3D), but the more central DSpd-2-GFP region retained a clear organisation, with several spoke-like projections of DSpd-2-GFP emanating outward into the PCM from a central ring (Figure 3D). We conclude that DSpd-2-GFP is part of a scaffold-like structure that forms around the centrioles and that retains some macromolecular structural integrity even in the absence of MTs.

### DSpd-2 and Cnn are incorporated into the PCM exclusively around mother centrioles

**Figure 4.**
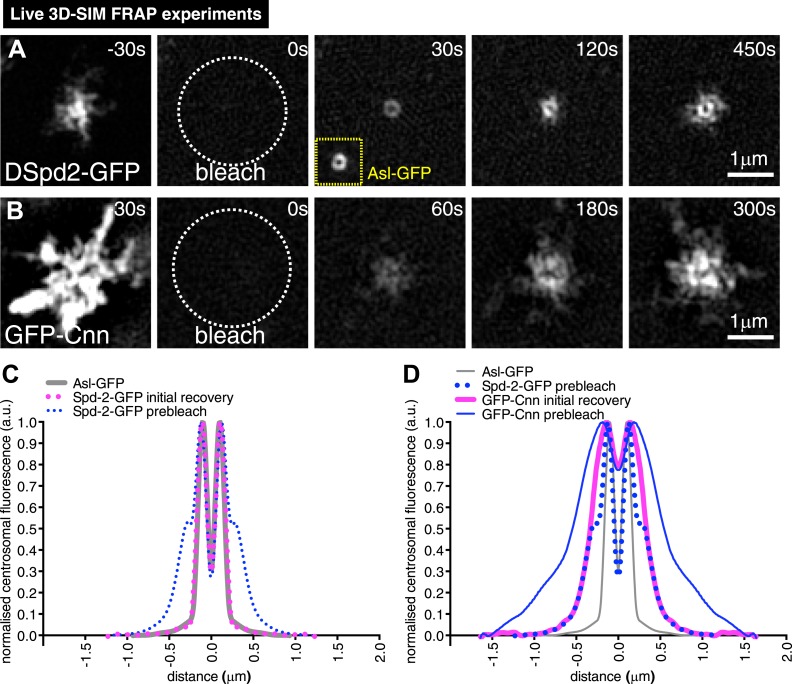
3D-SIM FRAP analysis of DSpd-2-GFP and GFP-Cnn behaviour at centrosomes. (**A** and **B**) 3D-SIM images show the dynamic behaviour of DSpd-2-GFP (**A**) and GFP-Cnn (**B**) at centrosomes in living *Drosophila* embryos after FRAP; time before and after photobleaching (t = 0) is indicated. (**A**) DSpd-2-GFP fluorescence initially recovers in a toroid shape around the centriole (**A**, t = 30 s), which has similar dimensions to unbleached Asl-GFP (yellow inset). The protein then moves slowly outwards, forming dynamic projections that spread away from the centriole. (**B**) GFP-Cnn fluorescence initially recovers in a broader region around the centrioles (t = 60 s in **B**), which has similar dimensions to the pre-bleached DSpd-2-GFP signal (t = −30 s in **A**). (**C**) Graph compares the average prebleached (dotted blue line) and initial recovery (dotted pink line) profiles of DSpd-2-GFP to the average unbleached profile of Asl-GFP (grey line); all profiles were normalized so that their peak value is equal to 1. Note how the initial DSpd-2-GFP recovery profile is essentially identical to the un-bleached Asl-GFP profile. (**D**) Graph compares the average pre-bleached (solid blue line) and initial recovery (solid pink line) profiles of GFP-Cnn, to the average pre-bleached profile of DSpd-2-GFP (dotted blue line), and the average unbleached profile of Asl-GFP (grey line); all profiles were normalized so that their peak value is equal to 1. Note how the initial GFP-Cnn recovery profile is very similar (particularly in the more peripheral regions), to the pre-bleached DSpd-2-GFP profile, but is quite distinct from the pre-bleached Asl-GFP profile. See also Video 2. **DOI:** http://dx.doi.org/10.7554/eLife.03399.010

In our 3D-SIM images, we noticed that DSpd-2 and, to a lesser extent, Cnn both seemed to emanate from a single toroidal structure that we presume contains the mother centriole (arrows, Figure 3A,C). To test if this structure was really the source of the centrosomal DSpd-2 and Cnn, we modified our 3D-SIM system to enable us (to our knowledge for the first time) to combine 3D-SIM with FRAP (‘Materials and methods’). This analysis revealed that after photobleaching DSpd-2-GFP fluorescence initially recovered in a toroidal pattern (t = 30 s, Figure 4A) and then moved slowly outwards on dynamic projections (t = 120 s to t = 450 s, Figure 4A; Video 2A). The initial toroidal distribution was very similar to that previously reported for Asl, one of the several proteins that form a toroid specifically around the mother centriole (Mennella et al., 2013) (compare the recovering DSpd-2-GFP signal at t = 30 s in Figure 4A to the unbleached Asl-GFP signal shown in the inset; also compare the average profile of Asl-GFP to the average initial recovery profile of DSpd-2-GFP, Figure 4C). Thus, DSpd-2-GFP is initially incorporated into the PCM by binding sites that are tightly concentrated around only one of the two centrioles—almost certainly the mother—and the spatial distribution of these sites extensively overlaps with the distribution of Asl.

**Video 2.** Super-resolution 3D-SIM FRAP experiments reveal the differences between how DSpd-2 and Cnn are incorporated into the PCM. (Related to Figure 4). All Videos shown here are maximum intensity projections of image stacks. Live cell SD-SIM illustrating the dynamic behaviour of DSpd-2-GFP (**A**) and GFP-Cnn (**B**) at centrosomes in *Drosophila* embryos. The employed OMX Blaze 3D-SIM system enables sub-diffraction live cell imaging at high frame rates with ∼two-fold better xy- and z-resolution compared to conventional microscopy. Time before and after photobleaching (t = 0 s) is shown at the top right of each panel. Note how, prior to photobleaching, GFP-Cnn has a broader distribution that DSpd-2-GFP within the PCM. Immediately after photobleaching, DSpd-2-GFP fluorescence recovers in the shape of a toroid around the centriole, supporting our conclusion that Asl, which is distributed as a toroid around the centriole, is the major recruiter of DSpd-2 to centrosomes. In contrast, GFP-Cnn fluorescence recovers in a broader region around the centrioles, supporting our conclusion that DSpd-2 is the major recruiter of Cnn to centrosomes. **DOI:** http://dx.doi.org/10.7554/eLife.03399.011

Interestingly, when we performed 3D-SIM FRAP with GFP-Cnn, the fluorescence initially recovered in the central region of the PCM (t = 60 s, Figure 4B), but this region was significantly broader than the toroidal region in which DSpd-2-GFP initially recovered (see t = 30 s, Figure 4A). Moreover, centrosomal GFP-Cnn fluorescence recovered more slowly than that of DSpd-2-GFP (compare Figure 1F to Figure 1—figure supplement 2M). Thus, although the centrosomal binding sites for DSpd-2 and Cnn appear to be concentrated around the mother centriole, they do not precisely overlap and they recruit DSpd-2 and Cnn at different rates, strongly suggesting that DSpd-2 and Cnn are not incorporated into the PCM together as part of the same complex.

### Asl is required for the efficient recruitment of DSpd-2 to mother centrioles

**Figure 5.**
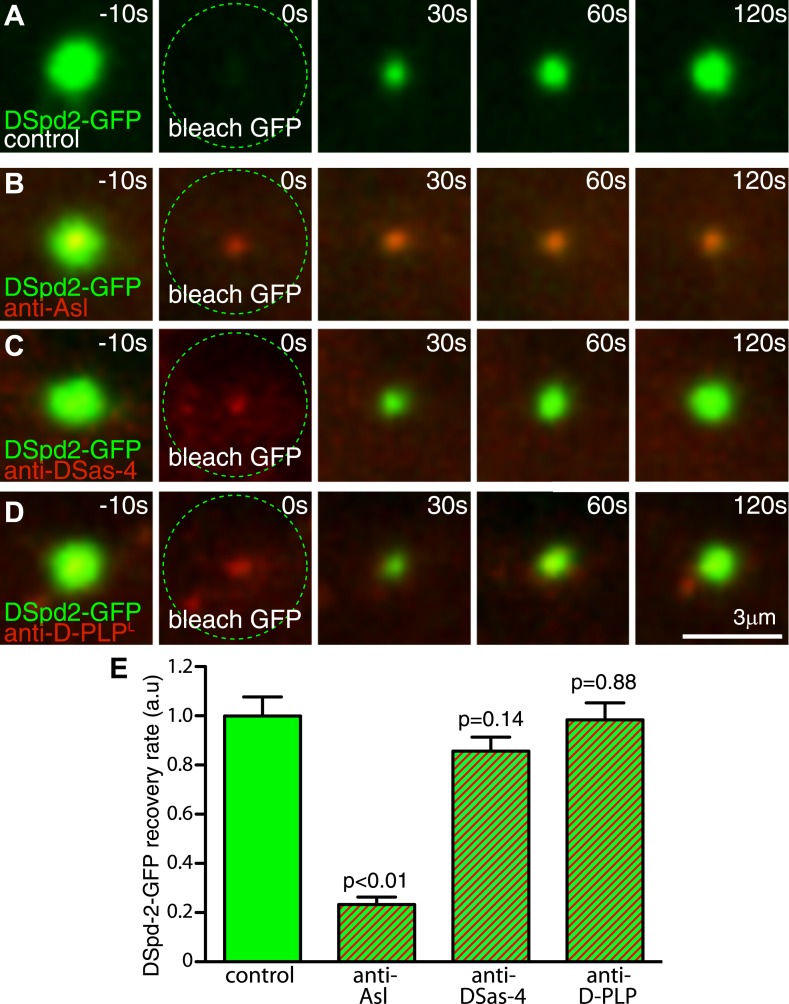
Inhibiting Asl function strongly perturbs DSpd-2 incorporation into the PCM. Images (**A**–**D**) show results from FRAP experiments monitoring how DSpd-2-GFP (green) incorporation into the PCM is affected by inhibiting the function of various centriole-associated components with injected Texas-red-labelled antibodies (red—as indicated). The antibodies bind to their cognate protein at centrosomes close to the injection site (**B**–**D**), but not to those far from the injection site (**A**), which therefore act as internal controls. (**E**) Graph displaying the initial recovery rate of DSpd-2-GFP fluorescence at centrosomes bound with antibodies (as indicated below the graph) relative to the centrosomes not bound by antibodies (control). The rate of recovery was calculated by measuring the gradient of the initial linear phase of recovery that occurred over the first 60 s after photobleaching. Note how anti-Asl antibodies reduce the rate of DSpd-2-GFP incorporation into the PCM by ∼75%, but that anti-DSas-4 or anti-D-PLP antibodies have little or no effect. Error bars = standard error. See also Video 3. **DOI:** http://dx.doi.org/10.7554/eLife.03399.012

**Figure 6.**
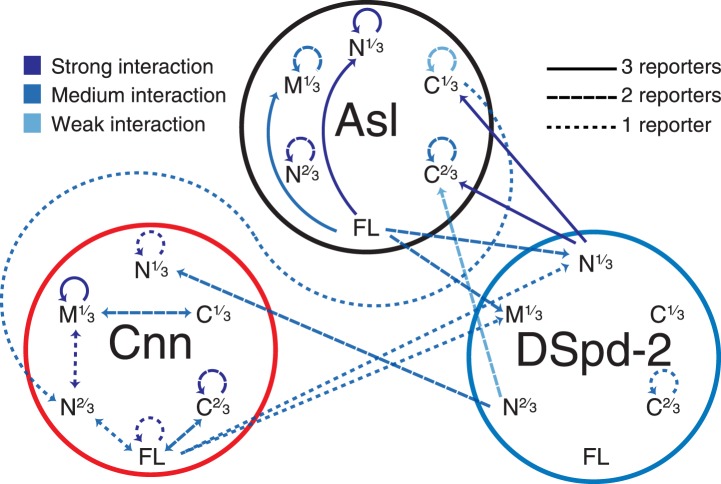
A yeast-two-hybrid analysis examining the interactions between Asl, DSpd-2, and Cnn. A schematic summary of the yeast two-hybrid interactions observed between Asl, DSpd-2, and Cnn. The shades of the lines indicate the strength of the interactions observed: strong (dark blue), medium (blue), and weak (light blue). The characteristics of the lines indicate the number of different positive reporter assays for each interaction: solid line (3/3 assays), large dashed line (2/3 assays), and small dashed line (1/3 assays). Arrows point from bait to prey fragments—double-headed arrows indicate that the interaction scored positive with either fragment as bait or prey. See Figure 6—source data 1. **DOI:** http://dx.doi.org/10.7554/eLife.03399.014 10.7554/eLife.03399.015Figure 6—source data 1.A yeast-2-hybrid analysis testing the interactions between various Asl, DSpd-2, and Cnn fragments.(Related to Figure 6) A yeast-2-hybrid analysis was carried out using various bait and prey fragments of each protein (as indicated in columns **A** and **B**). Three different reporters were tested—His (columns **C**–**F**), Ade (columns **G** and **H**), and LacZ (column **I**). Interaction levels are indicated as strong, medium, weak, or none. All combinations of baits and preys were tested, but only those that scored positive in at least one assay are shown.**DOI:** http://dx.doi.org/10.7554/eLife.03399.015

We wondered how DSpd-2 might be recruited to the toroidal structure surrounding the mother centriole. The centriole proteins DSas-4, Asl, and D-PLP all localize in a toroid pattern around mother centrioles (Fu and Glover, 2012; Mennella et al., 2012) and have all been implicated in PCM recruitment, suggesting that they could help recruit DSpd-2. Because DSas-4 and Asl mutants lack centrioles (Basto et al., 2006; Blachon et al., 2008), we tested this possibility by inhibiting the function of these proteins in embryos using antibody-injection (Conduit et al., 2010). Anti-Asl antibodies bound to centrosomes and reduced the rate of DSpd-2-GFP incorporation into the PCM by ∼75%, whereas anti-DSas-4 and anti-D-PLP antibodies had little effect (Figure 5)—although these antibodies bound to centrioles/centrosomes and at least partially disrupted the function of their cognate protein (Basto et al., 2008; Novak et al., 2014; unpublished data). These observations are consistent with the previous finding that Asl is required for the centrosomal localization of DSpd-2 (Giansanti et al., 2008). Moreover, our yeast two-hybrid (Y2H) analysis revealed several strong direct interactions between Asl and DSpd-2 (Figure 6, Figure 6—source data 1). Although we cannot be certain that Asl and DSpd-2 interact directly in vivo, collectively our data indicate that Asl has an important, and potentially direct, role in recruiting DSpd-2 to mother centrioles in fly embryos.

**Video 3.** The rate of DSpd-2-GFP incorporation into the PCM is reduced in embryos injected with anti-Asl antibodies. (related to Figure 5). All Videos shown here are maximum intensity projections of image stacks. These videos show the fluorescence recovery of DSpd-2-GFP (green) at centrosomes in embryos that were injected with fluorescently labelled antibodies (red) against Asl (**A**, **B**), DSas-4 (**C**), or D-PLP (**D**). Time before and after photobleaching (t = 0 s) is shown at the top right of (**D**). An example of a centrosome in an embryo injected with anti-Asl antibodies that was located far from the injection site and so received a low concentration of the antibody is shown in **(A**); these centrosomes acted as internal controls. Panels (**B**–**D**) show examples of centrosomes located close to the injection sites of antibodies against Asl (**B**), DSas-4 (**C**), and D-PLP (**D**); these centrosomes received a high concentration of each antibody. Note that DSpd-2GFP fluorescence recovered at a much slower rate at centrosomes that bound anti-Asl, whereas DSpd-2GFP fluorescence recovered at near normal rates at centrosomes that bound anti-DSas-4 or anti-D-PLP antibodies. **DOI:** http://dx.doi.org/10.7554/eLife.03399.013

### Asl and DSpd-2 are required for the efficient recruitment of Cnn to mother centrioles

We next addressed how Cnn might be recruited to mother centrioles. We previously showed that anti-DSpd-2 or anti-Asl antibodies, but not anti-D-PLP or anti-Sas-4 antibodies, inhibit the rate of Cnn incorporation into the PCM by ∼75% and ∼55%, respectively, suggesting that both DSpd-2 and Asl may have a role in recruiting Cnn to centrosomes (Conduit et al., 2010). We noticed, however, that the distribution of DSpd-2-GFP, but not Asl-GFP, around the mother centriole closely matched the distribution of the initial binding sites for GFP-Cnn, as revealed by our 3D-SIM FRAP experiments (compare the recovering GFP-Cnn signal at t = 60 s in Figure 4B to the unbleached DSpd-2-GFP signal at t = −30 s in Figure 4A; also compare the average profile of DSpd-2-GFP to the average initial recovery profile of GFP-Cnn, Figure 4D). This suggests that DSpd-2 may provide the major centrosomal binding site for Cnn in embryos.

**Figure 7.**
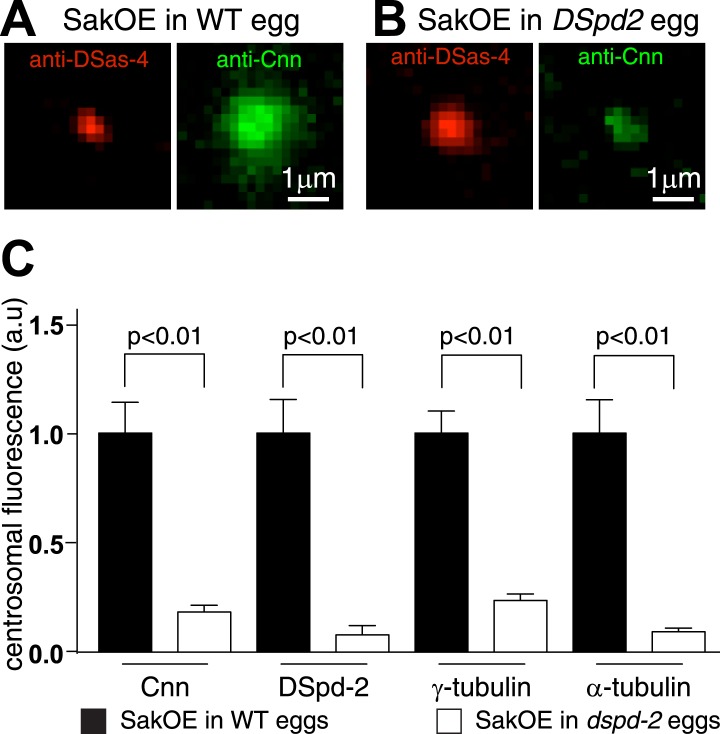
The centrosomal levels of Cnn are strongly reduced in eggs lacking DSpd-2. (**A** and **B**) Images show centrosomes co-stained with DSas-4 antibodies (red) and Cnn antibodies (green) in fixed eggs that either contained (**A**) or lacked (**B**) endogenous DSpd-2. As embryos lacking DSpd-2 fail in pronuclear fusion (Dix and Raff, 2007) (and so fail to develop), we induced the de novo formation of centrosomes in WT and *DSpd-2* mutant eggs by over-expressing Sak kinase (Peel et al., 2007; Rodrigues-Martins et al., 2007). The centrosomal levels of Cnn are dramatically reduced in the absence of DSpd-2. (**C**) The graph quantifying the centrosomal levels of Cnn, DSpd-2, γ-tubulin, and α-tubulin in Sak over-expressing eggs that either contained (black bars) or lacked (white bars) endogenous DSpd-2. The centrosomal levels of Cnn, γ-tubulin, and α-tubulin are dramatically reduced in the absence of DSpd-2. **DOI:** http://dx.doi.org/10.7554/eLife.03399.016

In support of this possibility, we found that the amount of Cnn localized to centrosomes in eggs was reduced by ∼80% in the absence of DSpd-2 (Figure 7) consistent with previous reports that the centrosomal localization of Cnn is perturbed in *dspd-2* mutant brain cells (Dix and Raff 2007; Giansanti et al., 2008). Moreover, our Y2H analysis revealed several strong direct interactions between DSpd-2 and Cnn but only one, much weaker, direct interaction between Cnn and Asl (Figure 6, Figure 6—source data 1). Although we cannot be certain that Cnn and DSpd-2 interact directly in vivo, collectively our data indicate that DSpd-2 has an important, and potentially direct, role in recruiting Cnn to mother centrioles in fly embryos, while Asl appears to have a more minor role.

### Cnn is not required to recruit DSpd-2 or Asl to centrosomes but is required to maintain high levels of DSpd-2 in the PCM

**Figure 8.**
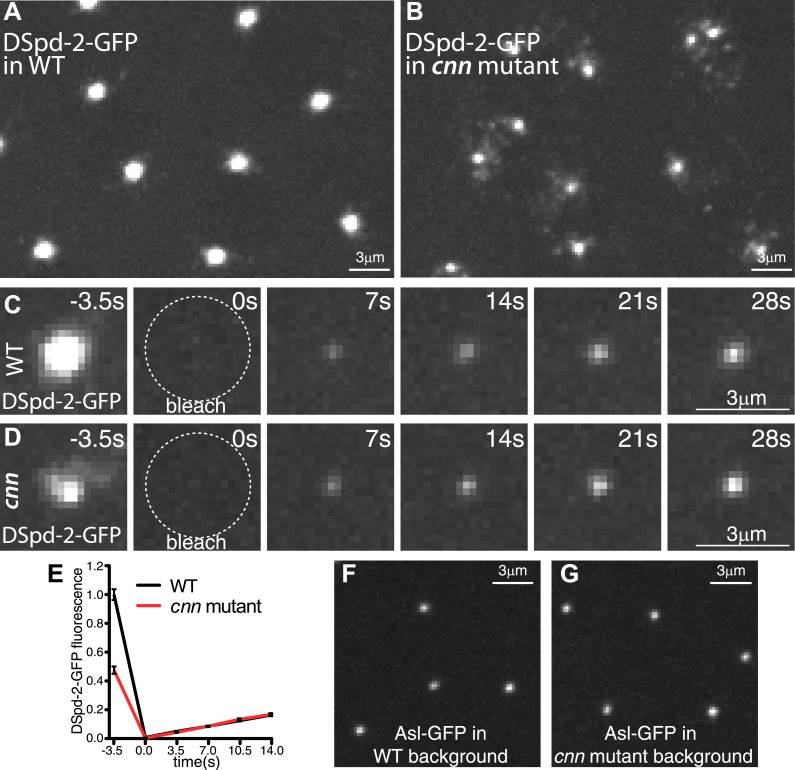
Cnn helps maintain DSpd-2 in the PCM. (**A** and **B**) Images show the localization of DSpd-2-GFP at centrosomes in either WT (**A**) or *cnn* mutant (**B**) embryos. (**C** and **D**) Images show the initial dynamic behaviour of DSpd-2-GFP at centrosomes in either WT (**C**) or *cnn* mutant (**D**) embryos; time before and after photobleaching (t = 0 s) is indicated. (**E**) Quantification of DSpd-2-GFP fluorescence recovery at centrosomes in either WT (black line) or *cnn* mutant (red line) embryos. The initial rate of DSpd-2-GFP fluorescence recovery is very similar in both WT and *cnn* mutant embryos, revealing that the initial incorporation of DSpd-2-GFP into the PCM is not dependent on Cnn. (**F** and **G**) Images show the localization of Asl-GFP at centrosomes in living embryos in the presence (**F**) or absence (**G**) of Cnn. The ability of Asl to localize efficiently in the absence of Cnn presumably explains why DSpd-2 can still be recruited to centrioles at normal rates in the absence of Cnn. Error bars = standard error. See Video 4. **DOI:** http://dx.doi.org/10.7554/eLife.03399.017

To investigate whether Cnn has a role in localizing DSpd-2 to centrosomes, we examined the distribution and dynamics of DSpd-2-GFP in *cnn* mutant embryos. Although there was a dramatic reduction in the amount of DSpd-2-GFP associated with centrosomes in the absence of Cnn (Figure 8A,B), FRAP experiments revealed that the initial rate of DSpd-2-GFP incorporation was unperturbed (Figure 8C–E). The localization of Asl-GFP (which appears to recruit DSpd-2) was also largely unperturbed in *cnn* mutant embryos (Figure 8F,G). The residual DSpd-2-GFP appeared to be more tightly associated with the centrioles in the absence of Cnn (Figure 8B), while the PCM fraction of the protein moved rapidly away from the centrioles in small ‘flares’ (Video 4). These observations suggest that Cnn is not required for the initial incorporation of DSpd-2 into the PCM, but is required for the proper maintenance of DSpd-2 within the PCM.

**Video 4.** The centrosomal localization of DSpd-2-GFP is perturbed in the absence of Cnn. (related to Figure 8). All Videos shown here are maximum intensity projections of image stacks. These videos illustrate the dynamic behaviour of DSpd-2-GFP at centrosomes in embryos where Cnn is present (**A**) or where Cnn is absent (**B**). Note how in the absence of Cnn, DSpd-2-GFP cannot properly spread out through the PCM and a haze of DSpd-2-GFP fluorescence, including small particles of DSpd-2-GFP, appears to be rapidly lost from the centrosomes. **DOI:** http://dx.doi.org/10.7554/eLife.03399.018

### Spd-2 and Cnn cooperate to recruit the mitotic PCM to centrioles

The experiments described above are consistent with the idea that DSpd-2 and Cnn form molecular scaffolds during mitosis that initially assemble around the mother centriole and then move slowly outward. Both proteins have been implicated in PCM recruitment, so we reasoned that such scaffolds might provide a platform on which other PCM proteins assemble during mitosis.

**Figure 9.**
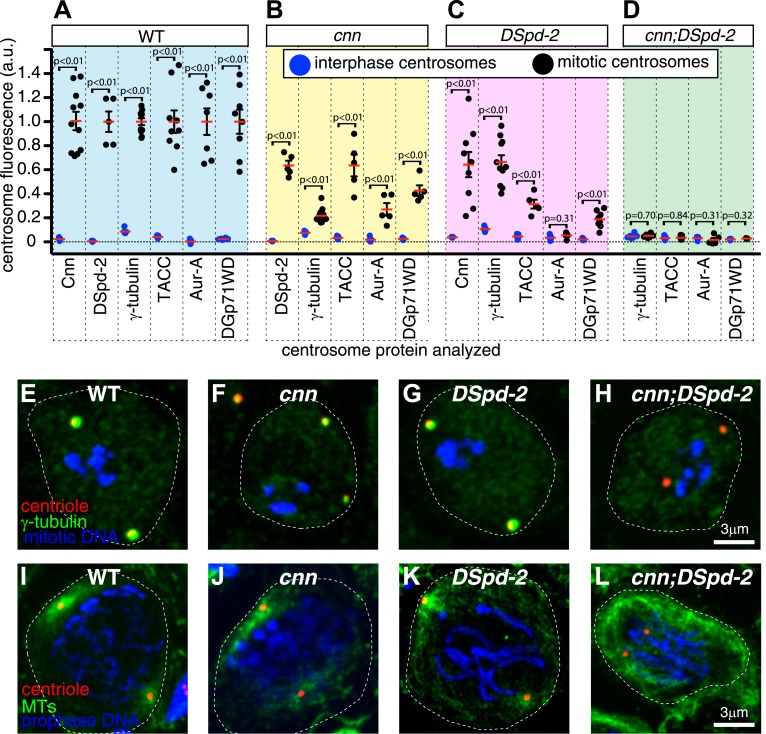
Cnn and DSpd-2 cooperate to recruit the mitotic PCM. (**A**–**D**) Graphs show the average fluorescence intensities of interphase (blue dots) and mitotic (black dots) centrosomes from either WT (**A**), *cnn* mutant (**B**), *dspd-2* mutant (**C**), or *cnn*;*dspd-2* double mutant (**D**) larval brain cells stained for various centrosomal proteins (as indicated below graphs). Each data-point represents the average centrosome value from one brain. The horizontal red bars indicate the average value of all the brains. All the PCM proteins are still partially recruited to centrosomes in the absence of Cnn or DSpd-2 (with the possible exception of Aurora A, which does not appear to be recruited in the absence of DSpd-2). The mitotic PCM levels do not rise above interphase levels in the absence of both Cnn and DSpd-2, indicating that centrosome maturation has been abolished. (**E**–**L**) Images show typical mitotic cells from either WT (**E** and **I**), *cnn* (**F** and **J**), *dspd-2* (**G** and **K**), or *cnn*;*dspd-2* double mutant (**H** and **L**) larval brain cells stained for the centriole marker Asl (red), mitotic DNA (phospho-histone H3, blue), and either the PCM marker γ-tubulin (green, **E**–**H**) or MTs (**I**–**L**, green). Error bars = SEM. See also Figure 9—figure supplements 1 and 2. **DOI:** http://dx.doi.org/10.7554/eLife.03399.019

To test this idea, we analyzed larval brain cells where centrioles organize almost no PCM or MTs during interphase but mature to organize large amounts of both during mitosis (Martinez-Campos et al., 2004; Rogers et al., 2008; Figure 9A). As shown previously, centrosome maturation in mitotic brain cells that lacked either Cnn (Megraw et al., 2001; Lucas and Raff, 2007) or DSpd-2 (Dix and Raff, 2007; Giansanti et al., 2008) was perturbed, but not abolished, and mitotic centrosomes organized levels of PCM that were significantly above those observed in interphase cells (Figure 9B,C,F,G). Remarkably, however, centrosome maturation appeared to be abolished in the mitotic brain cells lacking both Cnn and DSpd-2 (Figure 9D,H). This effect was specific to the loss of both Cnn and DSpd-2, as centrosomes in mitotic cells lacking either Cnn or DSpd-2 that also lacked the abundant PCM protein D-TACC could still partially mature (Figure 9—figure supplement 1). Thus, most PCM proteins appear to rely on Cnn and DSpd-2 for their recruitment to mitotic centrosomes.

**Figure 9—figure supplement 1.**
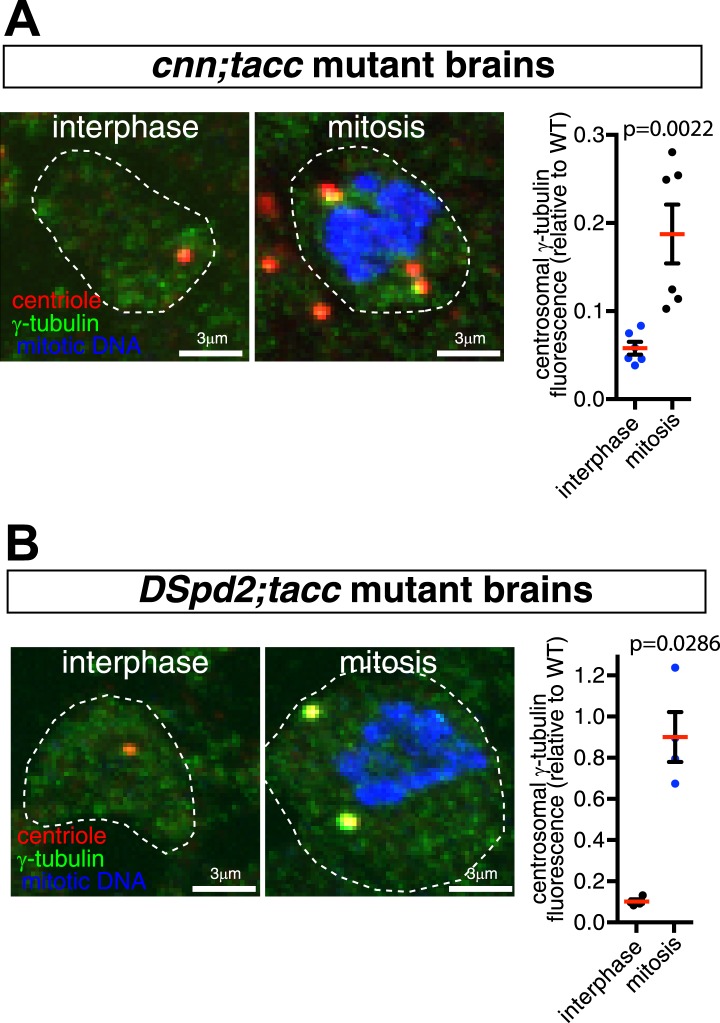
Centrosome maturation is not abolished in *cnn;tacc* or *dspd-2;tacc* double mutants. (**A** and **B**) Images and associated graphs show mitotic and interphase larval brain cells stained for γ-tubulin (green), the centriole marker Asl (red), and mitotic DNA (phospho-histone H3, blue) from either *cnn*;*tacc* double mutant (**A**) or *dspd-2*;*tacc* double mutant larvae (**B**). Graphs display the average fluorescent intensities of mitotic and interphase centrosomes (relative to a WT mitotic value of 1) stained for γ-tubulin. Each data-point represents the average centrosome value from one brain. Note how centrosomes still partially mature in each mutant combination, as the levels of γ-tubulin centrosomal fluorescence are significantly higher in mitotic cells than in interphase cells. **DOI:** http://dx.doi.org/10.7554/eLife.03399.020

**Figure 9—figure supplement 2.**
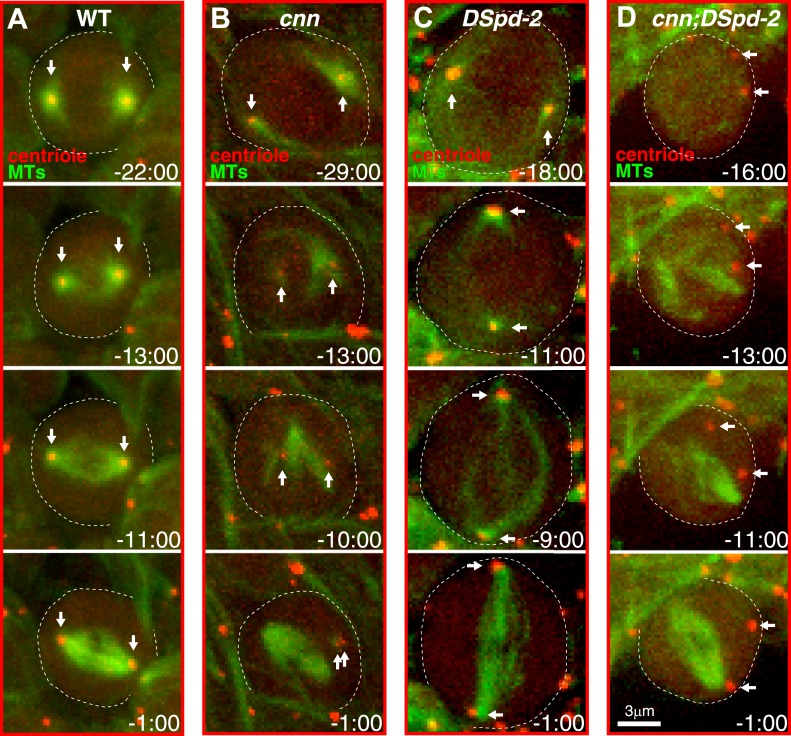
Centrosomes in *cnn*;*dspd-2* double mutants fail to organise MTs. Images show selected time-points from videos of either WT (**A**), *cnn* (**B**), *dspd-2* (**C**), or *cnn*;*dspd-2* double mutant (**D**) brain cells expressing the MT marker Jupiter-mCherry (pseudo-coloured green) and the centriole marker GFP-PACT (pseudo-coloured red); time before and after anaphase onset (t = 0 s) is indicated. Arrows indicate the position of the centrioles. The centrioles in WT cells, or *cnn* or *dspd-2* single mutant cells can organize MT asters, but no centriole-associated MTs can be detected in the *cnn*;*dspd-2* double mutants. **DOI:** http://dx.doi.org/10.7554/eLife.03399.021

To test whether centrioles in cells lacking both Cnn and DSpd-2 were really unable to form mitotic MTOCs, we analyzed MT behaviour. In fixed prophase cells (when centrosomal MT asters are most prominent), centrosomal asters were detectable in 100% of WT cells (Figure 9I; n = 11), 55% of cells lacking Cnn (Figure 9J; n = 58), 57% of cells lacking DSpd-2 (Figure 9K; n = 14), but in 0% of cells lacking both proteins (Figure 9L; n = 15). In live mitotic brain cells co-expressing the centriolar marker GFP-PACT and the MT marker Jupiter-mCherry (Figure 9—figure supplement 2; Video 5), centrosomes with MT asters were detectable in almost all cells lacking either Cnn (n = 18/18) or DSpd-2 (n = 24/25), but in almost no cells lacking both proteins (n = 1/24). We conclude that cells lacking both DSpd-2 and Cnn are unable to assemble centrosomal MTOCs during mitosis.

**Video 5.** Centrosomal MTOC activity is only abolished in cells lacking both Cnn and DSpd-2. (Related to Figure 9). All Videos shown here are maximum intensity projections of image stacks. These videos show the distribution of the centriole marker GFP-PACT (*pseudo-coloured red*) and the MT marker Jupiter-mCherry (*pseudo-coloured green*) in either WT (**A**), *cnn* (**B**), *dspd-2* (**C**), or *cnn;dspd-2* mutant neuroblasts as the cells progress through mitosis. Time before and after anaphase onset (t = 0 s) is shown at the top right of each panel. Note how the centrosomal MT asters can be observed in WT (**A**), *cnn* (**B**), and *dspd-2* (**C**) mutant neuroblasts, and these MT asters appear to contribute to spindle assembly. No centrosomal MT asters, however, can be observed in *cnn;dspd-2* double mutant neuroblasts and the spindle appears to form independently of centrosomes (**D**). **DOI:** http://dx.doi.org/10.7554/eLife.03399.022

## Discussion

Several hundred proteins are recruited to the PCM that expands around the centrioles during centrosome maturation in mitosis, but how so many proteins are organized into a functional mitotic centrosome has remained mysterious. Remarkably, we show here that the assembly of the mitotic PCM in flies appears to depend on just two proteins, Cnn and DSpd-2. Both proteins appear to form scaffolds that initially assemble around the mother centriole and then spread outward, forming a dynamic platform upon which most, if not all, other PCM proteins ultimately assemble. DSpd-2 and Cnn partially depend on each other for their centrosomal localization, and both proteins are required to ensure robust centrosome maturation. In the absence of one of these proteins, reduced levels of the other protein still localize around the centrioles and can support the partial assembly of the mitotic PCM. In the absence of both the proteins, mitotic PCM assembly appears to be abolished (Figure 9).

**Figure 10.**
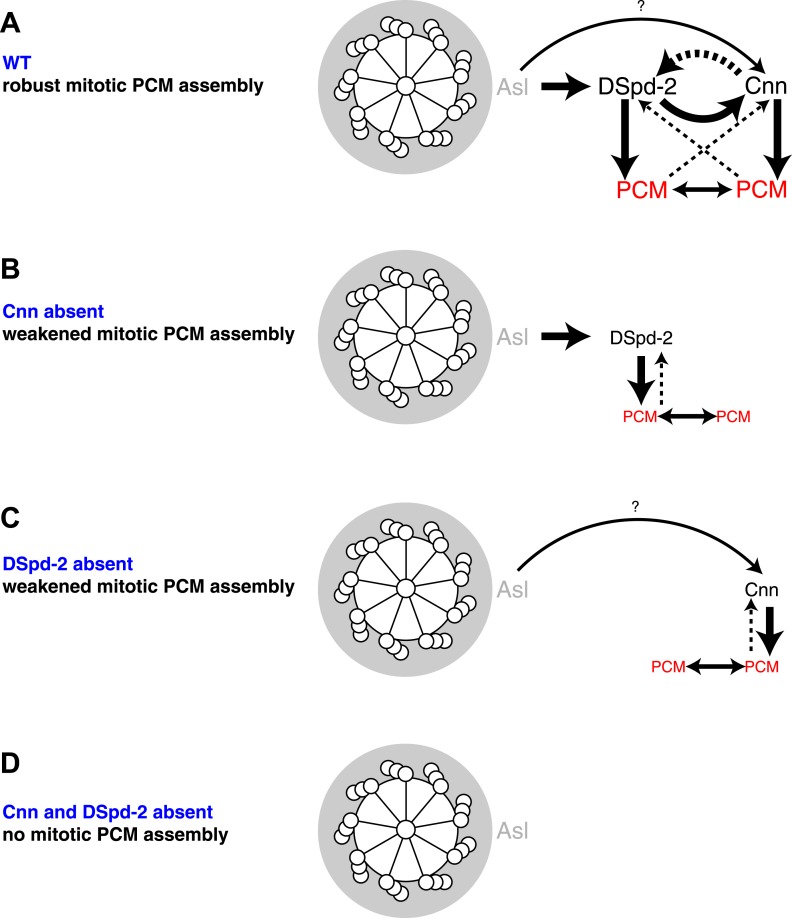
A model for mitotic PCM assembly in flies. Schematics illustrate a putative pathway of mitotic PCM assembly in a WT cell (**A**), or in cells lacking either Cnn (**B**), DSpd-2 (**C**), or both Cnn and DSpd-2 (**D**). A top view of the mother centriole is shown surrounded by a layer of Asl (grey); solid arrows represent recruiting interactions, dotted arrows represent maintaining interactions. Arrow thickness reflects the relative strength of the recruitment or maintenance, and the size of the text reflects the amount of protein localized at centrosomes. In WT cells (**A**), Asl has an important role in recruiting DSpd-2 to centrosomes, which in turn has an important role in recruiting Cnn; Cnn then has an important role in maintaining DSpd-2 at centrosomes. Thus, a positive feedback loop is generated where increasing amounts of DSpd-2 can recruit increasing amounts of Cnn, which can then maintain increasing amounts of DSpd-2. DSpd-2 and Cnn both independently recruit other PCM components (red), which themselves help support the PCM structure and can recruit further PCM components. In the absence of Cnn (**B**), Asl can still recruit DSpd-2 normally, but DSpd-2 cannot efficiently accumulate around the centrioles. The reduced levels of DSpd-2 recruit reduced levels of PCM. In the absence of DSpd-2 (**C**), an alternative pathway recruits reduced levels of Cnn. This pathway most likely involves Asl (as indicated here), as inhibiting Asl reduces the rate of Cnn incorporation into the PCM (Conduit et al., 2010) and Asl and Cnn appear to weakly interact in a Y2H analysis (Figure 6); other pathways, however, could also be involved. The reduced levels of Cnn recruit reduced levels of PCM. In the absence of Cnn and DSpd-2 (**D**), no mitotic PCM can be assembled. **DOI:** http://dx.doi.org/10.7554/eLife.03399.023

How are DSpd-2 and Cnn recruited to mother centrioles? Our results strongly suggest that in fly embryos Asl initially helps recruit DSpd-2 to centrioles and DSpd-2 then helps to recruit Cnn. Cnn does not appear to be required to recruit either Asl or DSpd-2 to centrosomes, but it is required to properly maintain DSpd-2 within the PCM. We speculate that this interaction between DSpd-2 and Cnn creates a positive feedback loop that drives the dramatic expansion of the PCM scaffold around mother centrioles during mitosis (Figure 10A). Although, we have identified direct interactions between Asl and DSpd-2 and between DSpd-2 and Cnn by Y2H, and the endogenous proteins can all co-immunoprecipitate with one another in fly embryo extracts (Conduit et al., 2010; Gopalakrishnan et al., 2011), we stress that we cannot be certain that these interactions are direct in vivo.

The requirement for Asl to initiate the mitotic recruitment of DSpd-2 and Cnn probably explains why these proteins are specifically recruited to mother centrioles. We recently showed that although Asl is essential for centriole duplication, it is not incorporated into daughter centrioles until they have passed through mitosis and matured into new mother centrioles (Novak et al., 2014), and Asl/Cep152 proteins mainly localize to mother centrioles in several species (Sir et al., 2011; Fu and Glover, 2012; Lawo et al., 2012; Mennella et al., 2012; Sonnen et al., 2012). The PCM appears to be preferentially associated with mother centrioles in many systems (Piel et al., 2000; Conduit et al., 2010; Wang et al., 2011). Our findings provide a potential explanation for why this is so, and raise the intriguing possibility that *all* mitotic PCM may be organized exclusively by mother centrioles.

Although DSpd-2 seems to be the major recruiter of centrosomal Cnn in embryos, there must be an alternative recruiter, as the centrosomal localization of Cnn is not abolished in the absence of DSpd-2 (Figure 7, Figure 9C). Asl is an attractive candidate as anti-Asl antibodies perturb Cnn recruitment to centrioles (Conduit et al., 2010) (although this could be an indirect consequence of their effect on DSpd-2 recruitment), and Asl and Cnn interact in our Y2H analysis. Moreover, human Cep152/Asl has a role in the centrosomal recruitment of human Cdk5Rap2/Cnn (Firat-Karalar et al., 2014). Interestingly, in flies this alternative pathway appears to be stronger in larval brain cells than in eggs/embryos: in the absence of DSpd-2, Cnn levels are reduced by only ∼35% in brains (Figure 9C) but by ∼80% in eggs (Figure 7C). Thus, the detail of mitotic PCM assembly pathway may vary between different cell types even in the same species.

Our data suggest that after DSpd-2 and Cnn have been recruited to centrioles, they rapidly assemble into scaffolds that then move slowly away from the centrioles. For Cnn, there is strong data indicating that scaffold assembly is regulated by phosphorylation. Cnn contains a phospho-regulated multimerization (PReM) domain that is phosphorylated by Polo/Plk1 in vitro and at centrosomes during mitosis in vivo (Dobbelaere et al., 2008; Conduit et al., 2014). Mimicking phosphorylation allows the PReM domain to multimerize in vitro and Cnn to spontaneously assemble into cytosolic scaffolds in vivo that can organize MTs. Conversely, ablating phosphorylation does not interfere with Cnn recruitment to centrioles, but inhibits Cnn scaffold assembly (Conduit et al., 2014). We speculate that, like Cnn, DSpd-2 can assemble into a scaffold and that this assembly is regulated in vivo so that it only occurs around mother centrioles. It remains unclear, however, whether DSpd-2 itself can form a scaffold, or whether it requires other proteins to do so.

It is striking that both DSpd-2 and Cnn exhibit an unusual dynamic behaviour at centrosomes. Both proteins incorporate into the PCM from the inside out, and are in constant flux, as the molecules that move slowly outward away from the centrioles are replaced by newly incorporated molecules close to the centriole surface (see Figure 1I). This inside out assembly is likely to have important consequences, as it means that the events close to the centriole surface, rather than at the periphery of the PCM, can ultimately regulate mitotic PCM assembly. This may be particularly important in cells where centrioles organise centrosomes of different sizes, as is the case in certain asymmetrically dividing stem/progenitor cells (Lesage et al., 2010; Nigg and Stearns, 2011; Pelletier and Yamashita, 2012). Fly neural stem cells, for example, use centrosome size asymmetry to ensure robust asymmetric division (Rebollo et al., 2007; Rusan and Peifer, 2007; Januschke et al., 2013), and there is strong evidence that new and old mother centrioles differentially regulate the rate of Cnn incorporation in these cells (Conduit and Raff, 2010). Moreover, mutations in human Cdk5Rap2/Cnn have been implicated in microcephaly (Bond et al., 2005), a pathology linked to a failure in neural progenitor cell proliferation, although the precise reason for this is unclear (Bond and Woods, 2006; Megraw et al., 2011).

Although DSpd-2 and Cnn have a major role in centrosome maturation, we stress that other PCM components are likely to make important contributions. Pericentrin, for example, has been implicated in PCM recruitment in several systems (Martinez-Campos et al., 2004; Zimmerman et al., 2004; Lee and Rhee, 2011; Lawo et al., 2012; Mennella et al., 2012; Kim and Rhee, 2014), and the fly homologue, D-PLP, forms ordered fibrils in cultured S2 cells that extend away from the centriole wall and support PCM assembly in interphase (Mennella et al., 2012). These centriolar fibrils, however, cannot explain how centrioles organize such a vastly expanded PCM matrix during mitosis, and D-PLP appears to have an important, but more minor, role in mitotic PCM assembly in vivo (Martinez-Campos et al., 2004). Nevertheless, proteins like D-PLP will certainly help recruit other PCM proteins and help form structural links within the PCM, thus strengthening the mitotic PCM matrix. The important distinction is that, in flies at least, most proteins, including the PCM fraction of D-PLP, are recruited into the PCM by an underlying PCM scaffold, whereas DSpd-2 and Cnn appear to form this scaffold.

Homologues of Asl, DSpd-2, and Cnn have been implicated in PCM assembly in many species (Pelletier et al., 2004; Gomez-Ferreria et al., 2007; Zhu et al., 2008; Haren et al., 2009; Barr et al., 2010; Cizmecioglu et al., 2010; Hatch et al., 2010; Joukov et al., 2010; Decker et al., 2011; Kim and Rhee, 2014), suggesting that the mechanism of mitotic PCM recruitment we identify here in flies may be conserved through evolution. To our knowledge, however, no PCM component has yet been shown to assemble from the inside out and to flux away from the centrioles in any other system. Nevertheless, although the precise molecular details will likely vary from cell type to cell type and from species to species, we suspect that this unusual dynamic behaviour of an underlying mitotic PCM scaffold will prove to be a general feature of mitotic centrosome assembly in many systems.

## Materials and methods

### Transgenic *Drosophila* lines

P-element-mediated transformation vectors were made by introducing a full-length DGp71WD or D-PLP cDNA (Kawaguchi and Zheng, 2004) into the Ubq-GFPCT Gateway vector (Basto et al., 2008). Transgenic lines were generated either by Genetic Services, Inc., Cambridge, MA, Bestgene, Inc., Chino Hills, CA or the Fly Facility in the Department of Genetics, Cambridge, UK. Other GFP, RFP, and mCherry fusions have been described previously: GFP-Cnn (Lucas and Raff, 2007), DSpd-2-GFP (Dix and Raff, 2007), Aur-A-GFP (Lucas and Raff, 2007), RFP-PACT (Conduit et al., 2010), Polo-GFP (Buszczak et al., 2006), Jupiter-mCherry (Callan et al., 2010), γ-tubulin-GFP (Hallen et al., 2008), DSas-4-GFP (Novak et al., 2014), and Asl-GFP (Blachon et al., 2008).

### Fly stocks

To examine the dynamics of DSpd-2-GFP and Asl-GFP at centrosomes, using both standard spinning disc confocal imaging and 3D-SIM imaging, we analyzed embryos from mothers expressing two copies of Ubq-DSpd-2-GFP in a *DSpd-2*^*Z35711*^/*DSpd-2*^*Df(3L)st-j7*^ hemizygous mutant background (Dix and Raff, 2007; Giansanti et al., 2008) or two copies of Asl-GFP (expressed from its endogenous promoter) in an *asl*^*mecd*^ mutant background (Blachon et al., 2008). For analysing the dynamics of γ-tubulin-GFP, AurA-GFP, DGp71WD-GFP, Polo-GFP, DSas-4-GFP, and D-PLP-GFP at centrosomes, we analyzed embryos from mothers expressing either two copies of γ-tubulin-GFP (expressed under the ncd promoter), Ubq-AurA-GFP, Ubq-DGp71WD-GFP, Polo-GFP (expressed under its endogenous promoter), DSas-4-GFP (expressed under its endogenous promoter) or one copy of Ubq-D-PLP-GFP in a WT background. For analysing the dynamics of GFP-Cnn at centrosomes using 3D-SIM imaging, we analyzed embryos from mothers expressing two copies of Ubq-GFP-Cnn in a *cnn*^*f04547*^/*cnn*^*HK21*^ hemizygous mutant background. For comparing the dynamics of DSpd-2-GFP in a WT background to the dynamics of DSpd-2-GFP in the absence of Cnn, we analyzed embryos from mothers expressing one copy of pUbq-DSpd-2-GFP in a WT background and embryos from mothers expressing one copy of pUbq-DSpd-2-GFP in a *cnn*^*HK21*^*/cnn*^*df(2R)BSC306*^ hemizygous mutant background. For comparing the localization of Asl-GFP in a WT background to the localization of Asl-GFP in the absence of Cnn, we analyzed embryos from mothers expressing one copy of pUbq-Asl-GFP in a WT background and embryos from mothers expressing one copy of pUbq-Asl-GFP in a *cnn*^*f04547*^/*cnn*^*HK21*^ hemizygous mutant background.

For examining PCM recruitment in larval brain cells lacking Cnn, DSpd-2 or Cnn, and DSpd-2, we analyzed *cnn*^*f04547*^/*cnn*^*HK21*^ hemizygous mutants, *dspd-2*^*Z35711*^/*dspd-2*^*Df(3L)st-j7*^ hemizygous mutant, or *cnn*^*f04547*^/*cnn*^*HK21*^; *dspd-2*^*Z35711*^/*dspd-2*^*Df(3L)st-j7*^ double hemizygous mutant larval brains, respectively.

For examining the behaviour of MTs in living larval brain cells, we analyzed brains expressing one copy of Ubq-GFP-PACT and one copy of Jupiter-mCherry (expressed under its endogenous promoter) in either a WT, *cnn*^*f04547*^/*cnn*^*HK21*^ hemizygous mutant, *dspd-2*^*Z35711*^/*dspd-2*^*Df(3L)st-j7*^ hemizygous mutant, or *cnn*^*f04547*^/*cnn*^*HK21*^; *dspd-2*^*Z35711*^/*dspd-2*^*Df(3L)st-j7*^ double hemizygous mutant background.

### Antibodies

For immunofluorescence analysis, we used the following antibodies: rabbit anti-Cnn (1:1000) (Lucas and Raff, 2007), rabbit anti-DSpd-2 (1:500) (Dix and Raff, 2007), mouse anti-γ-tubulin (1:500; GTU88, Sigma-Aldrich, St. Louis, MO), rabbit anti-D-TACC (1:500) (Gergely et al., 2000), rabbit anti-DGp71WD (1:500) (Vérollet et al., 2006), rabbit anti-AurA (1:500) (Barros et al., 2005), mouse anti-α-tubulin (1:1000; DM1α; Sigma-Aldrich), guinea-pig anti-Asl (1:500) (this study), and anti-PhosphoHistoneH3 (mouse, 1:2000, AbCam, UK or rabbit, 1:500, Cell Signalling Technology, Danvers, MA). Secondary antibodies were from Molecular Probes (Invitrogen, Carlsbad, CA): Alexa Fluor 488, 568, and 647 (all used at 1:1000).

For antibody injection experiments, we used rabbit anti-Asl (aa665–995), rabbit anti-DSas-4 (aa1–260), and rabbit anti-D-PLP (aa683–974) affinity purified antibodies. We also tested rabbit anti-D-PLP antibodies raised against aa1805–2137, which are predicted to also recognise the N-terminus of the short D-PLP isoform (aa8–350), and found that these antibodies, like the rabbit anti-D-PLP (aa683–974) antibodies, did not significantly perturb the DSpd-2-GFP incorporation (data not shown).

### Dynamic analysis of GFP-fusion proteins and image analysis

FRAP experiments were carried out as described previously (Conduit et al., 2014). Photobleaching was carried out in S phase, which is when mitotic PCM is most actively recruited in these rapidly cycling embryos. We used ImageJ to calculate the fluorescence profile of each centrosome at each time-point. We first scaled the images so that each pixel was split into 25 (5 × 5) pixels in order to increase the resolution of our radial profiling. We then calculated the centre of mass of the centrosome by thresholding the image and running the ‘analyze particles’ (centre of mass) macro on the most central Z plane of the centrosome. We then centred concentric rings (spaced at 0.028 μm and spanning across 3.02 μm) on this centre and measured the average fluorescence around each ring (radial profiling). After subtracting the average cytosolic signal and normalising, so the peak intensity of the pre-bleached image was equal to 1, we mirrored the profiles to show a full symmetric centrosomal profile. For each time-point, an average distribution from at least 10 centrosomes was calculated.

For analysing DSpd-2-GFP recovery in the centre and periphery of the PCM, we bleached centrosomes at the start of S-phase and then again 3 minutes later (still in S-phase); we measured the fluorescence recovery using radial profiling, as described above. The central PCM measurements were calculated as the average fluorescence intensity of 5 measurements taken between 0.028 μm and 0.14 μm, from the centre of the centrosome. The peripheral PCM measurements were calculated as the average fluorescence intensity of 5 measurements taken between 0.62 mm and 0.73 μm from the centre of the centrosome. Ten centrosomes from 10 embryos were analyzed; values were averaged to produce each data point. The cytosolic signal was subtracted before plotting the recovery graphs. To examine AurA-GFP and Polo-GFP recovery in the centre and periphery of the PCM, the original AurA-GFP and Polo-GFP FRAP data were re-analyzed by measuring the recovery in the same regions as for the DSpd-2-GFP analysis.

### Analysis of GFP-fusion expression levels

We fixed embryos in methanol, homogenized 50 embryos per genotype in 100 μl sample buffer, and ran either 5 μl or 10 μl on NuPAGE 3–8% Tris-acetate pre-cast gels (Life Technologies, Carlsbad, CA). The proteins were transferred onto nitrocellulose membrane and loading was initially checked using Ponceau staining. The membrane was then blocked and probed with antibodies against the protein in question and against the GFP.

### Super-resolution 3D structured illumination microscopy

Living embryos were imaged at 21°C on a DeltaVision OMX V3 Blaze microscope (GE Healthcare, UK) equipped with a 60x/1.42 oil UPlanSApo objective (Olympus), 405 nm and 488 nm and 593 nm diode lasers and sCMOS cameras (PCO). 3D-SIM image stacks were acquired with 5 phases 3 angles per image plane and 0.125μm z-distance between sections. The raw data was computationally reconstructed with SoftWoRx 6.0 (Applied Precision) using Wiener filter settings 0.002 and channel specifically measured optical transfer functions to generate a super-resolution 3D image stack with a lateral (x-y) resolution of 100-130 nm (wavelength-dependent) and an axial (z) resolution of ∼300 nm (Schermelleh et al, 2008). For two colour images, Images from the different color channels were registered with alignment parameter obtained from calibration measurements with 0.2 μm diameter TetraSpeck beads (Life Technologies) using the OMX Editor software. Images were processed using SoftWorx software (GE Healthcare). Images shown are maximum intensity projections of several z-slices. When analysing the effect of MT de-polymerisation, embryos were first injected with 1 mM colchicine solution and imaged 20–60 min later.

To perform 3D-SIM FRAP, we utilized the software development kit (SDK) from GE Healthcare. This allowed us to create a custom acquisition sequence that first acquired a single Z-stack in 3D-SIM, then performed single or multiple spot photobleaching (using the standard OMX galvo scanner TIRF/photo-kinetics module), and then performed time lapse imaging in 3D-SIM mode.

The centrosomal profiles were calculated in a similar way to that described above, except that the concentric rings for Asl-GFP, DSpd-2-GFP, and GFP-Cnn were spaced at 0.0055 μm, 0.011 μm, and 0.0109 μm and spanned across 1.86 μm, 3.28 μm, and 3.28 μm, respectively. For generating the average 3D-SIM profiles for Asl-GFP, DSpd-2-GFP, and GFP-Cnn, we averaged profiles from 11, 24, and 15 centrosomes, respectively.

### Antibody injections

Affinity-purified antibodies were covalently coupled to Texas Red, as described previously (Gergely et al., 2000). Antibodies were injected at the start of a mitotic cycle, and embryos were observed on the Spinning Disk confocal system described above. Centrosomes were bleached in pairs: one centrosome located close to the injection site (experimental) and one centrosome located far from the injection site (control). Between 2 and 3 centrosome pairs were bleached per embryo, with an average of 7 embryos injected for each antibody, and the data were collated. The average initial rate of DSpd-2-GFP incorporation at control and experimental centrosomes was compared using a paired Student's *t* test.

### Yeast two-hybrid

Bait and prey fragments were cloned, introduced into yeast, and tested for interactions as described previously (Conduit et al., 2014). For the baits, fragments encoding the N-terminal, middle, and C-terminal thirds of the proteins were cloned, along with fragments encoding the N-terminal two-thirds, C-terminal two-thirds, and the full-length protein. For the preys, smaller ∼200 aa fragments and larger combinations of these fragments, including the full-length protein, were cloned.

### Fixed brain analysis

For the analysis of centrosomal fluorescence levels of PCM components, third instar larval brains were dissected and incubated in 100 mM colchicine in Schneider's medium, Sigma-Aldrich for 1 hr at 25°C. Colchicine treatment de-polymerizes the MTs and prevents centrosome ‘rocketing’ in *cnn* mutants (Lucas and Raff, 2007), allowing a more accurate quantification of PCM recruitment. The brains were then fixed in paraformaldehyde containing 100 mM PIPES, 1 mM MgSO_4_, and 2 mM EGTA pH 6.95 for 5 min at room temperature, washed in PBS and then 45% and 60% acetic acid, squashed under a coverslip, post-fixed in methanol, washed in PBT, and then stained with the appropriate antibodies. Images were collected on an Olympus FV1000 scanning confocal microscope using a 60×, 1.4 NA oil objective and maximum intensity projections were made. For each brain at least five images containing multiple cells in both mitosis (as shown by positive Phospho-Histone H3 staining) and interphase were collected. At least fivebrains were imaged for each mutant and staining combination and an average of 40 mitotic and 37 interphase centrosomes were measured per brain. Centrosome fluorescence was calculated by measuring the total fluorescence in a boxed region around the centrosome and subtracting the local cytoplasmic background fluorescence. The average value of all the centrosomes from a single brain was used for each data point. The average value of these data points for mitotic and interphase cells were compared using a Mann–Whitney test. For the analysis of MT asters, the cells were fixed and stained as above, but were not pre-treated with colchicine. A cell was scored as positive if at least 1 centrosome had detectable astral MTs.

### Live brain analysis

Third instar larval brains were dissected and either semi-squashed under a coverslip or mounted whole in Schneider's medium and then imaged on the Perkin Elmer Spinning Disk confocal system described above. Cells were filmed progressing from interphase/prophase through mitosis. A cell was scored as positive if at least 1 centrosome had detectable astral MTs.
