# Supplementary material for: A molecular mechanism of mitotic centrosome assembly in Drosophila
Source: eLife. 2014 Aug 22;3:e03399. doi: 10.7554/eLife.03399 (PMC4175739; doi:10.7554/eLife.03399)
Supplement: Related texts

# Article commentary: Decision letter

**DOI:** 10.7554/eLife.03399.024

**Affiliations:** The Gurdon Institute, United Kingdom

eLife posts the editorial decision letter and author response on a selection of the published articles (subject to the approval of the authors). An edited version of the letter sent to the authors after peer review is shown, indicating the substantive concerns or comments; minor concerns are not usually shown. Reviewers have the opportunity to discuss the decision before the letter is sent (see review process). Similarly, the author response typically shows only responses to the major concerns raised by the reviewers.

Thank you for sending your work entitled “Asl, DSpd-2 and Cnn cooperate to assemble the mitotic PCM exclusively around mother centrioles” for consideration at *eLife*. Your article has been favorably evaluated by Tony Hunter (Senior Editor) and 3 reviewers, one of whom is a member of our Board of Reviewing Editors.

The Reviewing Editor and the other reviewers discussed their comments before we reached this decision, and the Reviewing Editor has assembled the following comments to help you prepare a revised submission.

The combination of 3D super-resolution imaging and FRAP has given interesting insights into how the PCM may be generated. This shows that DSpd-2 is initially incorporated into a tight toroid that coincides with the localization of the outer centriole wall and subsequently moves outward, whereas Cnn is initially incorporated into a broader structure that coincides with the full localization pattern of DSpd-2 (and other PCM proteins including D-PLP, Dgrip71WD and gamma-tubulin), from which it then moves outwards. These data are solid but the analysis of how these proteins are recruited is more tenuous. In particular the proposed pathway that Asl recruits D-Spd2, which forms a scaffold that recruits CNN, with partial feedback between D-Spd2 and CNN, is only one possible interpretation of the data and stronger evidence is required to make this conclusion. The reviewers were convinced by the evidence that Asl recruits D-Spd2 but not that D-Spd2 forms a matrix. Moreover, the yeast two-hybrid analysis does not stand well by itself since ambiguity remains over whether interactions are direct or not, and whether the interactions are conserved in their physiological setting. Similarly, the antibody injection results cannot be used as evidence that DSpd2 binds directly to CNN.

More experimental data will be required to address the following points:

1) More direct assays are required for the protein interactions between D-Spd2, CNN and Asl, or the conclusions must be toned down.

2) An important omission is that the authors should analyse the behaviour of D-PLP, because they have previously implicated it in PCM assembly, and because the human homologue has also been shown to have an important role.

3) The authors rely on transgenic flies expressing key centrosome proteins tagged with fluorescent proteins where expression levels may alter their dynamics and/or their distribution. This is particularly relevant when overexpressed since it has been well established that the overexpression of centrosome protein can modulate centrosome size. The authors should show data concerning the relative expression levels of the fusion proteins and mention how this may affect their results and interpretation.

4) The authors argue that DSpd-2 and Cnn form a lattice. Is this an inherent property of these two proteins or do the structures observed require MTs? Their linear extension could be consistent with them binding to MTs. Do they observe the same morphology in presence of nocodazole for example?

Minor comments (from Reviewer 2):

The authors would need to be more precise, throughout the manuscript, in what they mean by PCM, and the relative effects on PCM recruitment in different mutants. From this reviewer's perspective, there seems to be two steps in PCM recruitment, one in which PCM is recruited in close proximity to the centriole (i.e. toroidal appearance), the other in which the PCM moves from that location towards the periphery allowing more PCM to be recruited near the centriole and so forth. The generic use of PCM recruitment does not seem appropriate.

Several observations and key conclusions have been previously reported by the authors and other groups. For instance, it was known that DSpd-2 localization at the centrosome requires Asl activity and is independent to D-PLP, Cnn, -tubulin, DGrip91 and D-TACC. Also it was known that the centrosomal localization of Cnn is dependent on DSpd-2. The authors need to focus on their novel findings.

In Figure4A-B, the authors compared the central region of the DSpd-2-GFP and GFP-Cnn at 30s and 60s post-bleach respectively. Is there a reason why they are not both compared at 30s? Is 60s the first time point authors could detect recovery signal from GFP-Cnn, if so, why the recovery so much slower than DSpd-2-GFP considering authors proposed that Cnn is recruited to centrosome mostly by DSpd-2.

In Figure S1L, the authors claimed D-PLP-GFP normalized recovery profiles were wider than the pre-bleached profile, but did not give a clear explanation for it. This should be clarified.

The time scale in Figure 2E and 2G is much shorter than Figure 2B, can authors provide a longer recovery profile to show the extent of the recovery?

The authors report an extensive overlap between the distribution of DSpd-2-GFP and mCherry-Cnn (Figure 3B). The authors should provide quantitative data

The sample authors pointed (arrowhead) in Figure 3B did not support the claim they made in terms of DSpd-2-GFP, which did not extend outwards as far as mCherry-Cnn since the pointed area is at the edge of the Cnn positive area.

I do not think I saw any mention of Figure 3C in the text. Also in Figure 3B the area in blowups need to be indicated in original image.

# Reply: Author response

**DOI:** 10.7554/eLife.03399.025

*The combination of 3D super-resolution imaging and FRAP has given interesting insights into how the PCM may be generated. This shows that DSpd-2 is initially incorporated into a tight toroid that coincides with the localization of the outer centriole wall and subsequently moves outward, whereas Cnn is initially incorporated into a broader structure that coincides with the full localization pattern of DSpd-2 (and other PCM proteins including D-PLP, Dgrip71WD and gamma-tubulin), from which it then moves outwards. These data are solid but the analysis of how these proteins are recruited is more tenuous. In particular the proposed pathway that Asl recruits D-Spd2, which forms a scaffold that recruits CNN, with partial feedback between D-Spd2 and CNN, is only one possible interpretation of the data and stronger evidence is required to make this conclusion. The reviewers were convinced by the evidence that Asl recruits D-Spd2 but not that D-Spd2 forms a matrix. Moreover, the yeast two-hybrid analysis does not stand well by itself since ambiguity remains over whether interactions are direct or not, and whether the interactions are conserved in their physiological setting. Similarly, the antibody injection results cannot be used as evidence that DSpd2 binds directly to CNN*.

We were delighted with the generally positive tone of the comments, and I describe below how we have revised the manuscript in light of these comments/suggestions.

The major concern of the reviewers was that our putative recruitment pathway was tenuous. The evidence for this pathway is strong, as it is based on several lines of data: 1) Anti-Asl antibodies specifically suppress DSpd-2 recruitment, while anti-DSpd-2 antibodies specifically suppress Cnn recruitment; 2) Cnn, but not Asl, localisation is dramatically reduced in embryos lacking DSpd-2; 3) Neither DSpd-2 nor Asl recruitment is effected in embryos lacking Cnn, but DSpd-2 cannot be properly maintained at centrosomes. Moreover, the endogenous proteins all co-immunoprecipitate from embryo extracts (Conduit et al., 2010) and we can detect strong direct interactions between Asl and DSpd-2 and between DSpd-2 and Cnn in Y2H assays. Nevertheless, we agree that there are other ways of interpreting this data so we have now toned down our statements on this point throughout the manuscript.

The reviewers were convinced that Asl recruits DSpd-2, but not that DSpd-2 forms a matrix. While our previous study provided compelling evidence that Cnn forms a matrix (Conduit et al., Dev. Cell, 2014) we agree that this is less clear for DSpd-2. We now provide additional super-resolution data on Cnn and DSpd-2 behaviour in colchicine treated embryos that we think strengthens the argument for a DSpd-2 scaffold. Nevertheless, this is not conclusive, so we have toned down our conclusions throughout the text, and we explicitly state that it is unclear whether DSpd-2 itself can form a scaffold.

The reviewers raised some concerns about the physiological relevance of the Y2H interactions, and points out that the antibody injection experiments do not address whether DSpd-2 interacts directly with Cnn. We agree and now explicitly state that we cannot be certain that these interactions are direct in vivo.

More experimental data will be required to address the following points:

*1) More direct assays are required for the protein interactions between D-Spd2, CNN and Asl, or the conclusions must be toned down*.

They requested that we either provide more direct assays to confirm the interactions between these proteins or that we tone down our conclusions on this point. As described above, we have toned down our conclusions accordingly.

*2) An important omission is that the authors should analyse the behaviour of D-PLP, because they have previously implicated it in PCM assembly, and because the human homologue has also been shown to have an important role*.

The reviewers requested that we analyse the behaviour of D-PLP as this protein has been implicated in PCM recruitment. We now describe our analysis of D-PLP behaviour in more detail, and explain why the quantitative analysis of this protein is complicated by the presence of a slowly turning over fraction in the centriole and a rapidly turning over fraction in the PCM (legend to Figure 1–figure supplement 2). As we now state, it is clear that D-PLP in the PCM does not function like DSpd-2 or Cnn as a component of an underlying PCM scaffold during mitosis.

*3) The authors rely on transgenic flies expressing key centrosome proteins tagged with fluorescent proteins where expression levels may alter their dynamics and/or their distribution. This is particularly relevant when overexpressed since it has been well established that the overexpression of centrosome protein can modulate centrosome size. The authors should show data concerning the relative expression levels of the fusion proteins and mention how this may affect their results and interpretation*.

The reviewers request data on the expression levels of the various GFP-fusions relative to their endogenous proteins. We now provide this data in Figure 1–figure supplement 1, and discuss its implications in the legend. While the overexpression of GFP-fusion proteins can lead to differences in the rate of protein binding to centrosomes (and we have shown that this is the case for some of these proteins; Conduit et al., Curr. Biol., 2010), this does not seem to effect the mechanism of how the proteins bind to centrosomes, and we have shown this to be the case for several of the proteins we analyse here, including GFP-Cnn (Conduit et al., Curr. Biol., 2010).

4) The authors argue that DSpd-2 and Cnn form a lattice. Is this an inherent property of these two proteins or do the structures observed require MTs? Their linear extension could be consistent with them binding to MTs. Do they observe the same morphology in presence of nocodazole for example?

The reviewers queried whether the DSpd-2 and Cnn lattices require MTs for their formation. This is an excellent question, and we now include a 3D-SIM analysis of DSpd-2 and Cnn behaviour in living embryos injected with colchicine to depolymerise the MTs. This analysis has been very informative. Although both proteins clearly associate with MTs in the peripheral region of the PCM, this is not the case in the central region. For DSpd-2 in particular, a clear structured lattice is still apparent in the central region even when the MTs have been depolymerised.

Minor comments (from Reviewer 2):

*The authors would need to be more precise, throughout the manuscript, in what they mean by PCM, and the relative effects on PCM recruitment in different mutants. From this reviewer's perspective, there seems to be two steps in PCM recruitment, one in which PCM is recruited in close proximity to the centriole (i.e. toroidal appearance), the other in which the PCM moves from that location towards the periphery allowing more PCM to be recruited near the centriole and so forth. The generic use of PCM recruitment does not seem appropriate*.

The reviewer points out that there appear to be two steps to PCM recruitment. We agree, although we were slightly confused as to whether the reviewer meant two phases to general PCM recruitment (i.e. interphase, where the PCM is recruited to a tight area around the centrioles [as shown by others], and mitosis, where the PCM is recruited to an expanded scaffold of DSpd-2 and Cnn that forms around the centrioles) or that there were two phases to the specific recruitment of DSpd-2 and Cnn (where both proteins are initially recruited to centrioles [step 1], and they then assemble into lattices that move outwards away from the centrioles [step 2]). In either case we have attempted to emphasise both points throughout the revised manuscript.

*Several observations and key conclusions have been previously reported by the authors and other groups. For instance, it was known that DSpd-2 localization at the centrosome requires Asl activity and is independent to D-PLP, Cnn, -tubulin, DGrip91 and D-TACC. Also it was known that the centrosomal localization of Cnn is dependent on DSpd-2. The authors need to focus on their novel findings*.

The reviewer pointed out that some of our observations had already been reported previously, and we now make sure we properly acknowledge where this is the case. In most of these instances, however, we feel that our new observations add important extra information. For example, the reviewer is right that Giansanti et al., (Curr. Biol., 2008) concluded that the centrosomal localisation of DSpd-2 was independent of D-PLP, Cnn, ©-tubulin, DGrip71 and D-TACC; our live cell studies now show, however, that although Cnn is not required to recruit DSpd-2 to centrosomes, it is required to maintain DSpd-2 at centrosomes, and this is central to our proposed pathway.

*In* *Figure4A-B**, the authors compared the central region of the DSpd-2-GFP and GFP-Cnn at 30s and 60s post-bleach respectively. Is there a reason why they are not both compared at 30s? Is 60s the first time point authors could detect recovery signal from GFP-Cnn, if so, why the recovery so much slower than DSpd-2-GFP considering authors proposed that Cnn is recruited to centrosome mostly by DSpd-2*.

The reviewer raises the important point that DSpd-2-GFP recovers faster than GFP-Cnn, and this is indeed why we show a first recovery time-point of 30s for the former and 60s for the latter. We are grateful to the reviewer for pointing this out, as we realise we did not explain this aspect of our model very well. Although DSpd-2 recruits Cnn, our data strongly suggests they are not recruited to the centrosome together as part of the same complex (hence the differential recruitment rates). We believe that DSpd-2 is recruited to centrosomes primarily by Asl; once recruited, DSpd-2 then becomes a platform for recruiting Cnn. We now clarify this important point.

*In Figure S1L, the authors claimed D-PLP-GFP normalized recovery profiles were wider than the pre-bleached profile, but did not give a clear explanation for it. This should be clarified*.

The reviewer asks for clarification of the D-PLP-GFP FRAP recovery curve. As discussed in point (2) above, we now provide much more information on this.

*The time scale in* *Figure 2E and 2G* *is much shorter than* *Figure 2B**, can authors provide a longer recovery profile to show the extent of the recovery?*

The reviewer points out that the time scale of Figure 2E and 2G is much shorter than Figure 2B and asks that we provide a longer recovery profile for E and G. These graphs show FRAP recovery curves for Polo-GFP, Aurora A-GFP and DSpd-2-GFP, respectively, and the reason for the different time scales is that Polo and Aurora A recover much more quickly than DSpd-2. Throughout this study we have concentrated on comparing initial recovery rates and distribution profiles and have not attempted to show full recovery curves or to calculate mobile/immobile fractions for all these proteins. This is because each protein has a unique behaviour and because the centrosomes grow in size, so these proteins accumulate throughout S-phase to different extents, making simple comparisons very difficult. We feel it would significantly distract from the main point of the paper if we were to provide and properly document a full description of the dynamic behaviour of all individual proteins we analyse here.

*The authors report an extensive overlap between the distribution of DSpd-2-GFP and mCherry-Cnn (**Figure 3B**). The authors should provide quantitative data*

The reviewer requests a more quantitative analysis of the overlap between the distribution of DSpd-2-GFP and mCherry-Cnn. Quantifying this in a meaningful way is problematic as these 3D-SIM images are all calculated reconstructions from multiple image stacks that, for technical reasons, have to be acquired sequentially (red first then green); the PCM is rapidly moving in these embryos making voxel by voxel comparisons unreliable, particularly in the crowded environment of the centrosome. What we think is clear from our analysis is that there is substantial (but not complete) overlap between the distributions of DSpd-2 and Cnn (at least by eyeball) in the central region of the PCM, but that Cnn strongly extends out into the more peripheral regions, where DSpd-2 is much weaker (although there is usually some DSpd-2 detectable in the peripheral regions that contain Cnn). We now explain this more clearly, and provide an additional Figure 3–figure supplement 1 that shows examples of line plots through a centrosome that we hope illustrate these points in a semi-quantitative manner (although we are not big fans of this type of analysis as they can be very subjective).

*The sample authors pointed (arrowhead) in* *Figure 3B* *did not support the claim they made in terms of DSpd-2-GFP, which did not extend outwards as far as mCherry-Cnn since the pointed area is at the edge of the Cnn positive area*.

The reviewer commented on whether the DSpd-2-GFP extended out as far as the mCherry-Cnn. There may have been a typo in this comment as it was a little difficult to understand exactly what the reviewer meant here. I hope that the changes described in our response to the above point address this issue.

Finally, your Features Editor, Peter Rodgers, wrote to us asking us to change the title, pointing out that most readers would not be familiar with the term PCM or with any of the protein names. We have now done this.
